# Reversing zinc dyshomeostasis and oxidative stress: A metallothionein 1-activating MXene/Zn^2+^ triple-action nanotherapeutic strategy for chronic non-bacterial prostatitis

**DOI:** 10.1016/j.bioactmat.2026.07.037

**Published:** 2026-08-21

**Authors:** Kailai Liu, Yuchen Zhang, Yanyao Gao, Yunhe Zheng, Yu Huang, Jiangchuan He, Ting Wang, Hanchao Zhou, Jinpeng Wen, Zhengye Sun, Keke Wang, Qiang Fu, Bin Song, He Wang, Lei Wang, Geng Zhang, Ke Wang

**Affiliations:** a School of Pharmacy, Health Science Center, Xi'an Jiaotong University, Xi'an, Shaanxi Province, 710061, China; b Department of Urology, Tangdu Hospital, Air Force Medical University, Xi'an, Shaanxi Province, 710038, China

**Keywords:** Chronic non-bacterial prostatitis, Zinc ion homeostasis, Ti_3_C_2_ MXene, Metallothionein 1, Targeted drug delivery

## Abstract

Chronic non-bacterial prostatitis (CNP), a prevalent and debilitating urological disorder affecting 8.4% of men aged 15–60 years, presents significant clinical challenges due to the paucity of targeted therapies and poor patient adherence. To address this unmet medical need, we developed an innovative multifunctional nanoplatform (QM (Zn) NPs) by integrating Ti_3_C_2_ MXene with a quercetin-zinc coordination complex (Que-Zn) for precision CNP therapy. This system leverages chondroitin sulfate (Chs)-mediated CD44 targeting to achieve selective accumulation in inflamed prostate tissue, thereby enhancing Zn^2+^ bioavailability while enabling co-delivery of MXene and Que-Zn therapeutic payloads. Upon localization, QM (Zn) NPs orchestrate a coordinated therapeutic cascade: MXene scavenges reactive oxygen species (ROS) *via* electron-deficient sites, while Que-Zn drives M1-to-M2 macrophage repolarization and facilitates Zn^2+^ cellular uptake. The accumulated intracellular Zn^2+^ critically upregulates metallothionein 1 (Mt1), activating the IKK/NF-κB/IκB axis to resolve inflammation and oxidative damage. Transcriptomic analysis unequivocally identified Mt1 as the pivotal mediator of Zn^2+^-driven microenvironment reprogramming. Notably, QM (Zn) NPs not only significantly alleviated pelvic pain by mitigating neuronal oxidative stress but also exhibited excellent biocompatibility. This work pioneers a targeted nano-theranostic strategy that synergistically restores zinc homeostasis, quenches ROS, and reprograms immune responses, thereby establishing a transformative paradigm for CNP management.

## Highlights

• CD44-targeted MXene/Zn^2+^ nanoplatform corrects prostate Zn^2+^ dyshomeostasis in CNP *via* Mt1 upregulation.
• Triple-action therapy: MXene scavenges ROS, Que-Zn repolarizes macrophages & delivers Zn^2+^ to activate IKK/NF-κB anti-inflammatory axis.
• Transcriptomics-confirmed Mt1-mediated microenvironment reprogramming alleviates neuronal oxidative stress and pelvic pain.

## Graphical abstract

**Figure:**
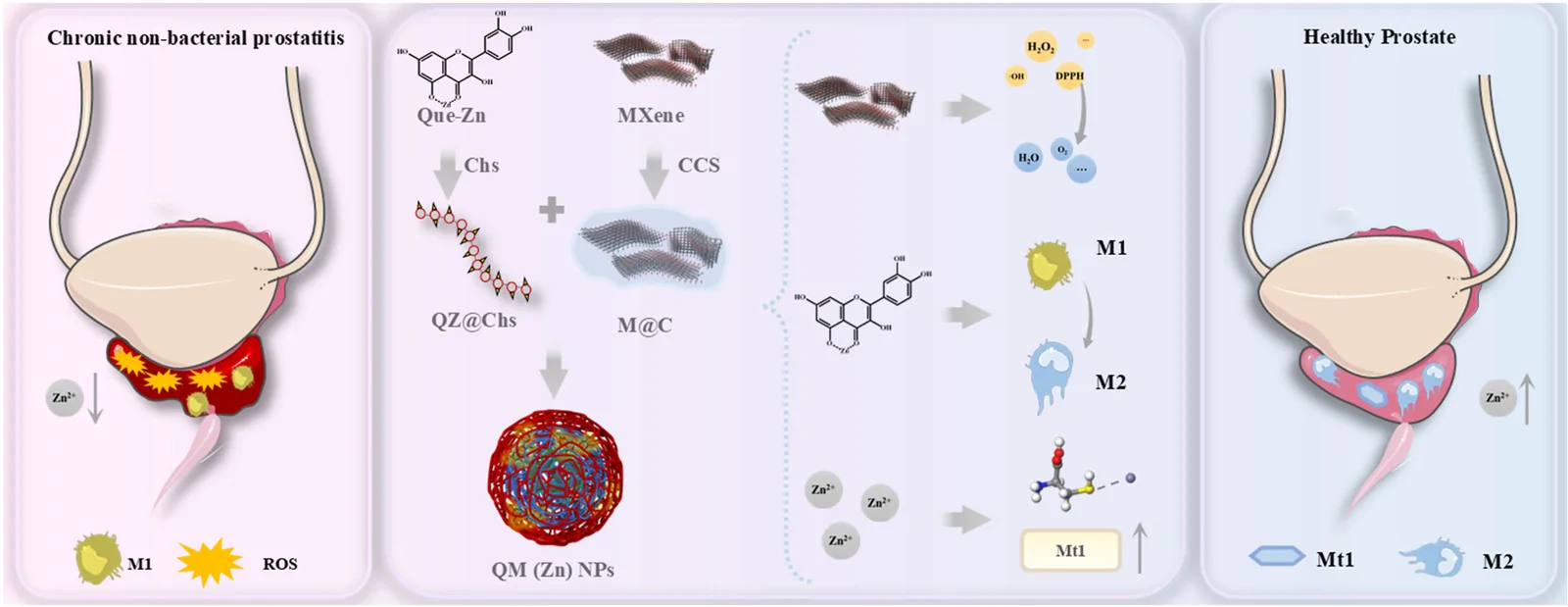

## 1 Introduction

**Scheme 1:**
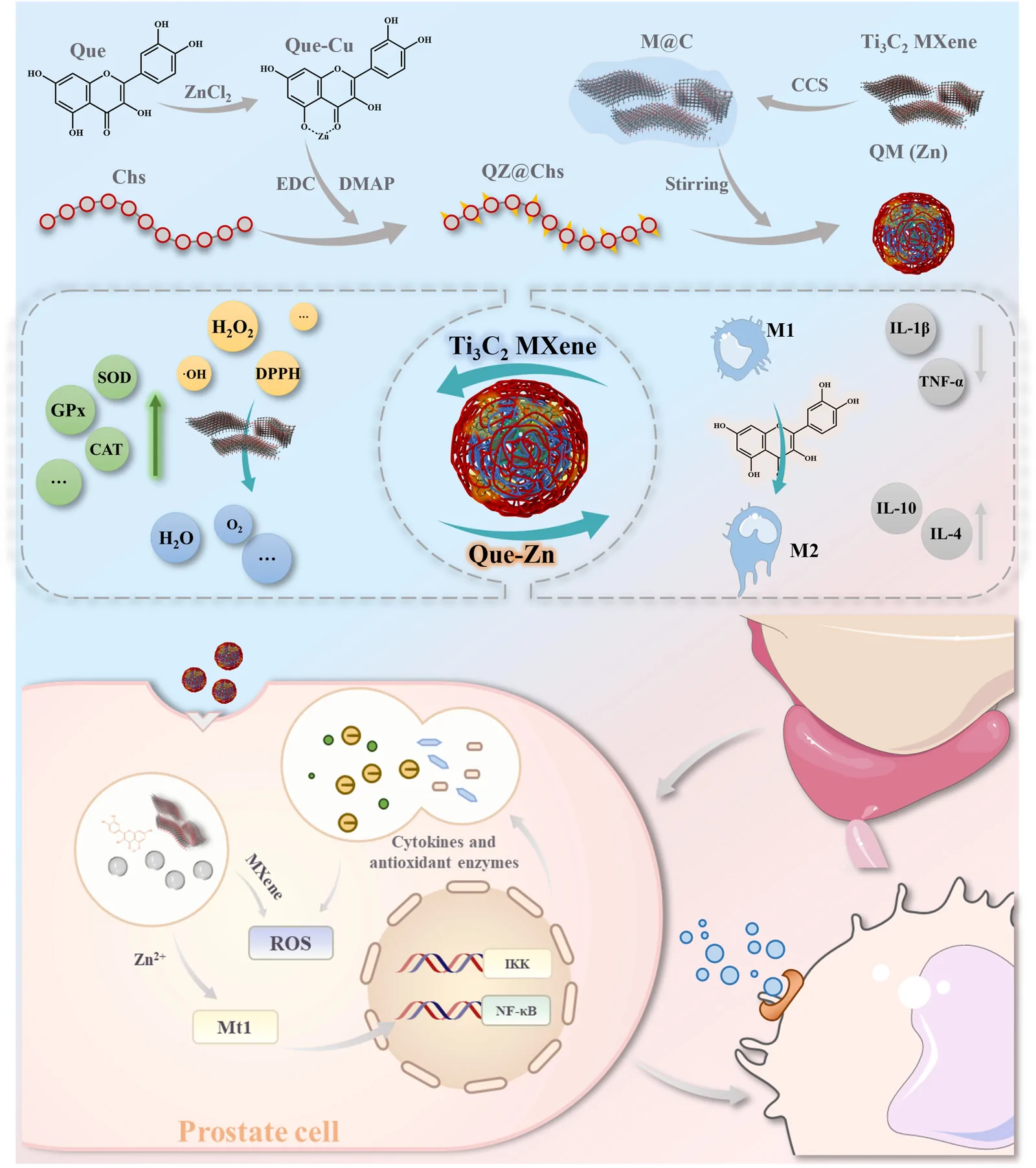
The schematic illustration of preparation, application, and therapeutical mechanism of QM (Zn) NPs on Chronic non-bacterial prostatitis.

Prostatitis ranks among the most prevalent conditions in the urology and men's outpatient clinics [1]. Epidemiological data [2] revealed that 8.4% of males aged 15-60 years are affected by prostatitis with 90% of case diagnosed as chronic non-bacterial prostatitis (CNP), also known as chronic pelvic pain syndrome (CPPS) [3]. CNP manifests through persistent pelvic/perineal pain, dysuria, diminished semen quality, and sexual dysfunction [[4], [5], [6], [7]]. Its chronic recurrent course significantly impairs patients' quality of life and psychosocial well-being [1,8]. Its multifactorial etiology involves three primary components: poor lifestyle habits (sedentary behavior, nocturia, urine retention, alcohol consumption, spicy diets), prostate-specific anatomical features inducing chronic blood stasis with consequent hypoxic microenvironments and redox imbalance [1], and altered zinc ion (Zn^2+^) homeostasis as a key pathogenic determinant [9]. These mechanisms collectively drive local free radical accumulation, inflammatory mediator release, and prostate microenvironmental disruption. Current clinical management primarily employs pharmacotherapy (antibiotics, α-blockers, muscarinic receptor antagonists, NSAIDs/analgesics) [10,11]. along with physical therapy modalities such as acupuncture, hyperthermia, magnetotherapy, and other physical therapy intervention [[12], [13], [14]]. Despite demonstrated efficacy, limitations including non-targeted drug delivery, inability to address multifactorial pathogenesis, lack of long-term efficacy and the invasiveness/repeated visits required for physical interventions progressively erode patient compliance and trust [15]. This frequently leads to treatment discontinuation, disease chronicity, and symptom recurrence, creating an urgent clinical imperative for novel targeted CNP therapeutics (see Scheme 1).

Mounting evidence in recent years has highlighted the prostate microenvironment as a critical focus in CNP research. Among the various factors involved in maintaining prostate homeostasis, zinc ion has attracted increasing attention because of its essential role in preserving normal prostate physiology. Several studies have reported the relationship of zinc ion concentration dynamics and prostate microenvironment alterations may affect progression and resolution of CNP [[16], [17], [18]]. Under physiological conditions, the prostate microenvironment maintains elevated Zn^2+^ concentrations essential for cellular stability and normal function [19]. During inflammatory conditions, several studies have reported decreased prostatic Zn^2+^ levels accompanied by disruption of the local microenvironment [20,21]. These accumulating evidence suggests an association between alterations in Zn^2+^ homeostasis and prostate microenvironmental imbalance [22]. Additionally, Zn^2+^ deficiency has been reported to be associated with persistent microenvironmental dysregulation and increased susceptibility to prostate pain [15]. Experimental studies have suggested that zinc supplementation may alleviate prostatic pain and improve certain CNP-related manifestations in preclinical models [[23], [24], [25]]. However, these findings have yet to be translated into clinical recommendations, and effective strategies for regulating zinc homeostasis within inflamed prostatic tissue remain lacking. Current Zn^2+^ delivery modalities, including oral preparations, local injections, and dietary supplements, lack precision. The development of targeted Zn^2+^ delivery systems for prostate epithelial cells in CNP management remains an unmet research priority. Crucially, the mechanistic basis of Zn^2+^ -mediated prostatitis alleviation remains poorly characterized [[26], [27], [28]]. Therefore, identifying zinc-related molecular targets may facilitate a better mechanistic understanding of the potential role of zinc homeostasis in prostatitis. Notably, Zn^2+^ concentration directly modulates expression levels of metallothionein 1 (Mt1), a key regulatory target [29]. Zn^2+^ binding to Mt1's cysteine-rich domain induces its expression and enhances antioxidant capacity [30]. Mt1 has been reported to participate in cellular antioxidant defense by regulating antioxidant enzymes, including superoxide dismutase (SOD) and catalase (CAT), thereby mitigating oxidative stress. Collectively, these findings raise the possibility that Zn^2+^ may alleviate prostatitis by modulating the prostate microenvironment through Mt1-related pathways, although this mechanism remains to be experimentally validated.

In previous research [31], a multifunctional nano-delivery system was developed for co-delivering Ti_3_C_2_ MXene with bifacial functionality and Cu^2+^ *via* quercetin. This system enhanced the antimicrobial capacity and improved the photodynamic properties of Ti_3_C_2_ MXene, enabling multimodal treatment of bacterial prostatitis. We also explored its potential for treating non-bacterial prostate. Consequently, we hypothesized that substituting Cu^2+^ with Zn^2+^ in this nano-delivery system might be efficacious in addressing CNP. This substitution was motivated by two key rationales: first, Zn^2+^ could improve the prostate microenvironment to facilitate pain relief; second, the inherent cascade enzyme activity of Ti_3_C_2_ MXene could provide potent antioxidant protection. Building upon this foundation, we constructed a multifunctional nano-delivery system capable of delivering Zn^2+^ and MXene to the prostate site for targeted CNP treatment. Specifically, we loaded zinc ions onto natural quercetin and subsequently grafting it onto chondroitin sulfate, while MXene nanosheets were dispersed using CCS to enhance their water solubility; finally, these two components were integrated to form the QM (Zn) nanodrug delivery system. This approach achieved targeted zinc delivery and enhanced bioavailability. System characterization confirmed that, the system enabled prostate tissue targeting through Chs-CD44 receptor mediation. The efficacy of this system in alleviating pain and ameliorating inflammation was validated through both cellular and animal experiments. Most notably, our findings revealed substantial variations in Mt1 expression levels before and after treatment, as evidenced through transcriptomics. These findings provide mechanistic evidence supporting the potential role of zinc homeostasis in CNP and contribute to a better understanding of zinc-mediated regulation of the prostate microenvironment. This pivotal discovery not only establishes the zinc-Mt1 axis as a master regulatory mechanism in CNP pathogenesis but also provides a pioneering theoretical framework for metal ion homeostatic nanotherapeutics in prostatitis management.

## 2 Results and discussion

### 2.1 Preparation and Characterization of QM (Zn) NPs

**Fig. 1:**
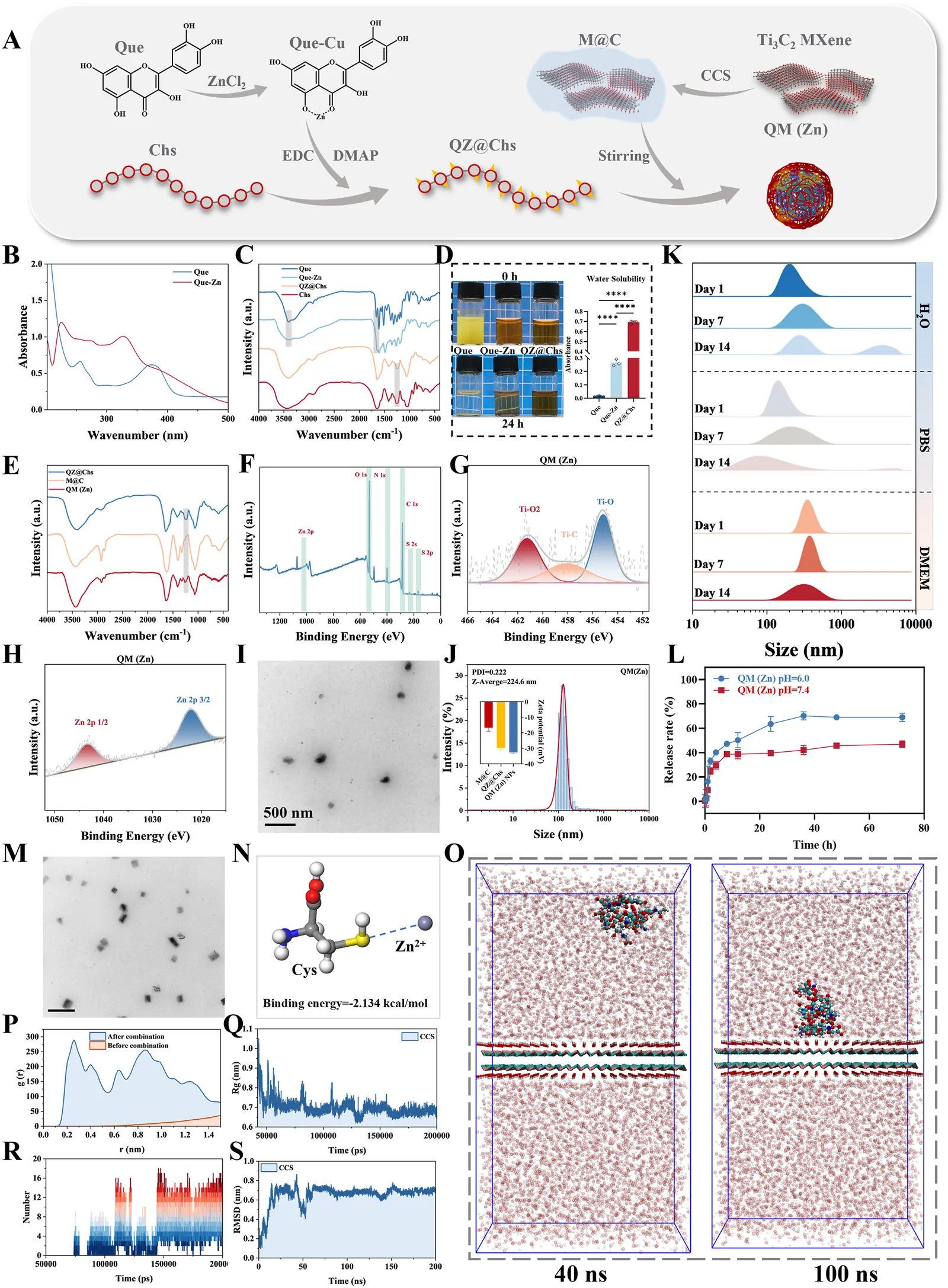
Preparation and Characterization of QM (Zn) NPs. **A**, The schematic of preparation of QM (Zn) NPs. **B**, The UV absorption spectrum of Que and Que-Zn. **C**, The FTIR spectrum of Que, Que-Zn, QZ@Chs and Chs. **D**, The water solubility of Que, Que-Zn, and QZ@Chs. n = 3. **E**, The FTIR spectrum of QZ@Chs, M@C and QM (Zn). **F**, The XPS survey spectrum of QM (Zn). **G**, The Ti high-resolution spectra of QM (Zn). **H**, The Zn high-resolution spectra of QM (Zn). **I**, The representative TEM image of QM (Zn) NPs. **J**, The size distribution of QM (Zn) NPs and zeta potential of QZ@Chs, M@C and QM (Zn) NPs. n = 3. **K**, The particle size distribution of QM (Zn) NPs under different treatments. **L**, The release profile of Que-Zn under acidic and neutral conditions. n = 3. **M**, The TEM image of Ti_3_C_2_ MXene release. Scale bar = 500 nm. **N**, The binding energy of cysteine sulfhydryl groups and Zn^2+^. **O**, The molecular dynamics simulation results of self-assembly of MXene and CCS. **P**, The radial distribution functions of free CCS and CCS bound to MXene. **Q**, The radius of gyration during the combination of CCS and MXene. **R**, The number of hydrogen bonds in the binding process of CCS and MXene. **S**, T root-mean-square deviation of CCS during the binding process of CCS and MXene. All values are expressed as mean ± SD. Statistical significance was determined using a one-way ANOVA with Tukey's multiple comparisons. ****P < 0.0001.

In light of the pivotal function of zinc ions in the prostate function, we engineered a nano delivery system co-loaded with zinc ions and MXene, drawing upon the methodologies established in prior studies. This system utilizes the natural flavonoid quercetin as a zinc ion carrier, with the preparation process delineated in Fig. 1A. In the initial phase, the quercetin-zinc complex (Que-Zn) was synthesized. UV-visible absorption spectroscopy (Fig. 1B) revealed characteristic absorption peaks for both free Que and Que-Zn, with observed peak shifts following zinc coordination attributable to altered electron cloud distribution in quercetin upon Zn^2+^ binding. Infrared spectroscopy (Fig. 1C) confirmed complex formation: the observation of the difference between Que and Que-Zn reveals a displacement of the telescopic vibrational peak of -OH from 3408 cm^−1^ (Que) to 3392 cm^−1^ (Que-Zn) upon coordination with zinc ions. The displacement of the telescopic vibrational peak of C=O from 1662 cm^−1^ (Que) to 1650 cm^−1^ (Que-Zn) is indicative of the formation of coordination bonds between Zn^2+^ and the oxygen atoms of -OH and C=O of quercetin (Fig. 1A). Subsequent grafting of Que-Zn onto Chs, a novel peak emerged in the IR spectrum at 1227 cm^−1^, attributed to C-O-C in the ester bond. Complementary NMR analysis (Fig. S1) revealed benzene ring protons of Que at 6.7-7.8 ppm in QZ@Chs, confirming successful conjugation. Water solubility was significantly enhanced through this grafting. As demonstrates visually and quantitatively in Fig. 1D, both Que exhibited low solubility in water and behaved as turbid liquids. Following an extended period of standing, the insoluble substances precipitated at the base of the bottle. Que-Zn exhibited a modest degree of water solubility, yet its solubility was limited. Consequently, the color of the remaining liquid (the “supernat”) underwent a substantial lightning after the standing period. However, the remarkable water solubility of QZ@Chs ensured that there was no substantial change in the color of the supernat before and after standing. This finding was further substantiated by the detection of solution absorbance (Fig. 1D).

Subsequently, Ti_3_C_2_ MXene was loaded onto carboxylated chitosan (CCS) to obtain M@C. The UV-vis absorption spectrum of Ti_3_C_2_ MXene (Fig. S2) exhibited characteristic absorption peaks in the near-infrared region, consistent with our previous studies. As demonstrated in prior study [31], CCS significantly enhanced the aqueous dispersion stability of Ti_3_C_2_ MXene in M@C. In addition, it was ascertained that the binding between Ti_3_C_2_ MXene and CCS is predominantly facilitated by hydrogen bonds. Subsequently, a combination of QZ@Chs and M@C was employed to synthesize QM (Zn) NPs. The characteristic peaks of QZ@Chs and M@C were discernible in the IR spectra of the nanoparticles depicted in Fig. 1E, validating successful nanoparticle assembly. Subsequently, the elemental compositions of the QM (Zn) NPs were analyzed using X-ray photoelectron spectroscopy (XPS). The presence of the elements carbon (C), oxygen (O), nitrogen (N), sulfur (S), zinc (Zn), and titanium (Ti) in QM (Zn) NPs confirmed the presence of QZ@Chs and Ti_3_C_2_ MXene, as demonstrated by the full-scan pattern in Fig. 1F and the fine pattern in Fig. 1G and H, and S3. The metal ion content of QM(Zn) NPs was quantified by ICP-MS, revealing a Zn loading of 2.8% (w/w) and a Ti content of 1.0% (w/w). As illustrated in Fig. 1I, the microscopic morphology of the QM (Zn) NPs was observed by TEM, revealing a particle morphology around 200 nm. This observation is attributed to hydrogen bonding and hydrophobic interactions, resulting in the encapsulation of Ti_3_C_2_ MXene within the interior, while QZ@Chs and CCS form the outer shell. The energy dispersive spectroscopy (EDS) elemental mapping images (Fig. S4) demonstrate the presence of the elements carbon (C), sulfur (S), zinc (Zn), and titanium (Ti) within the structure of the QM (Zn) NPs. The results of Fig. 1J demonstrate that the particle size of QM (Zn) NPs is 224.6 nm, which is consistent with the TEM results. The potential of QM (Zn) NPs is −30 mV, a property that facilitates their stable dispersion in solution. The aforementioned results collectively demonstrate a successful synthesis of QM (Zn) NPs.

The stability of a pharmaceutical agent is a critical determinant of their shelf life. To assess the stability of QM (Zn) NPs, we investigated their behavior in water, PBS, and DMEM solutions. As demonstrated in Fig. S5, Ti_3_C_2_ MXene sedimented completely, in all three solutions by day 7, whereas QM (Zn) NPs exhibited minimal sedimentation. Minor precipitation of QM (Zn) NPs was observed by day 14. Particle size analysis (Fig. 1K) revealed no significant change in QM (Zn) NPs across the solutions on day 7. By day 14, limited aggregation had occurred, forming some larger particles, but the majority remained stable. These finding indicate that QM (Zn) NPs can be stored in solution for over seven days. The therapeutic efficacy of a nano-delivery system depends critically on drug release. Fig. 1L demonstrates that QM(Zn) NPs exhibit enhanced Que-Zn release under acidic conditions, capable of releasing over 70% of the drug. This is attributed to the acid-induced destabilization of ester bonds. Such pH sensitivity facilitates drug release within the slightly acidic microenvironment of inflamed tissue. The observed initial release under neutral pH (7.4) can be attributed to the factor that a fraction of the Que-Zn complex is physically adsorbed on the nanoparticle surface, which rapidly desorbs and diffuses into the medium. Furthermore, TEM observations (Fig. 1M) indicate that the Ti_3_C_2_ MXene released upon nanoparticle disintegration retain their nanosheet structure, enabling sustained catalytic activity.

To complement experimental characterization and probe the fundamental interactions governing our nanoplatform, we integrated computational modeling with biology approaches. Since the metal-binding domain of Mt1 is composed of cysteine-rich motifs, the interaction between Zn^2+^ and thiol groups was first evaluated by DFT calculations using a cysteine model in Fig. 1N. The calculated binding energy (−2.134 kcal/mol) indicates a favorable interaction between Zn^2+^ and sulfur ligands. Importantly, previous isothermal titration calorimetry (ITC) studies have directly demonstrated the high-affinity and domain-specific binding of Zn^2+^ to metallothionein, providing thermodynamic evidence for Zn–Mt1 interaction [32]. This result theoretically validates our rationale for targeting Mt1 *via* Zn^2+^. Building upon our prior work demonstrating MXene-CCS association through hydrogen bonding, this study further conducted molecular dynamics simulations at the theoretical level (Fig. 1O). Given computational constraints with large molecular weights, partial MXene unit cells and representative CCS polysaccharide segments were selected for simulation. Over a 200 ns trajectory in aqueous solution, initially separated MXene and CCS gradually associated, with CCS stably adsorbing onto the MXene surface. Radial distribution function (RDF) analysis (Fig. 1P) revealed that free CCS primarily distributed around 1.5 nm, while MXene-bound CCS exhibited peaks at 0.4 nm and 0.9 nm. The radius of gyration results (Fig. 1Q) showed decreased and stabilized values after CCS-MXene complexation, indicating conformational contraction and enhanced stability. Hydrogen bond counts (Fig. 1R) and root mean square deviation (RMSD) values (Fig. 1S) both increased and stabilized upon complex formation. Collectively, these results demonstrate the spontaneous self-assembly tendency between CCS and MXene in aqueous environments and the stable complex formation.

### 2.2 Antioxidant activity of QM (Zn) NPs

**Fig. 2:**
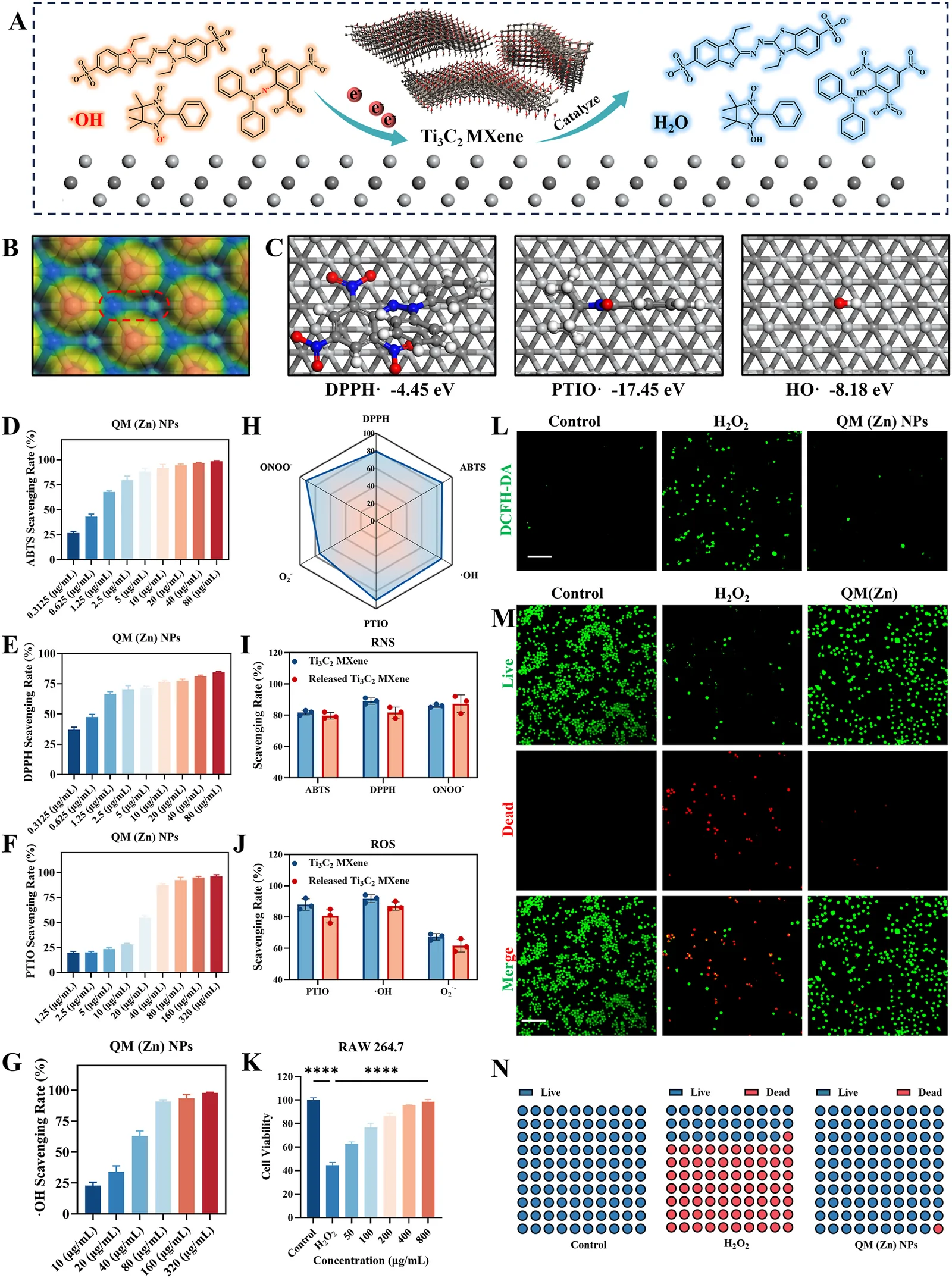
ROS/RNS-Scavenging Activities of QM (Zn) NPs. **A**, The schematic of scavenging RO/NS. **B**, The electron cloud density distribution of Ti_3_C_2_ MXene. **C**, The DFT calculations of the binding energies of Ti_3_C_2_ MXene and free radicals. **D**, The ABTS scavenging ratios of QM (Zn) NPs. n = 3. **E**, The DPPH scavenging ratios of QM (Zn) NPs. n = 3. **F**, The PTIO scavenging ratios of QM (Zn) NPs. n = 3. **G**, The ·OH scavenging ratios of QM (Zn) NPs. n = 3. **H**, The comparison of the scavenging ability of QM (Zn) NPs on different free radicals. **I**, The comparison of RNS capabilities. n = 3. **J**, The comparison of RNS capabilities. n = 3. **K**, The cell viability of RAW264.7 cells under H_2_O_2_ and QM (Zn) NPs treatment. n = 4. **L**, The representative ROS staining images of RAW 264.7 cells under H_2_O_2_ and QM (Zn) NPs treatment. Scale bar = 100 μm. **M**, The representative live/dead staining images of RAW 264.7 cells under H_2_O_2_ and QM (Zn) NPs treatment. Scale bar = 100 μm. **N**, Statistical results of fluorescence ratio. All values are expressed as mean ± SD. Statistical significance was determined using a one-way ANOVA with Tukey's multiple comparisons. ****P < 0.0001.

Prolonged oxidative stress is a primary contributor to numerous chronic diseases, many of which lack effective treatments, stemming from an imbalance between the production and clearance of reactive oxygen species (ROS) and reactive nitrogen species (RNS). Consequently, rapidly eliminating ROS/RNS and restoring redox homeostasis represents a pivotal step in treating such conditions. Given numerous reports highlighting MXene's potent ROS-scavenging ability (Fig. 2A), we evaluated the antioxidant capacity of both MXene and QM(Zn) NPs. Density functional theory (DFT) calculations of Ti_3_C_2_ MXene's electron cloud distribution (Fig. 2B) revealed numerous electron-deficient sites with low electron density in the central regions of surface Ti atoms, proposed as the reactive centers for ROS interaction. Supporting this, calculated binding energies between Ti_3_C_2_ MXene and three representative free radicals (Fig. 2C, based on ref [33]) were all negative, demonstrating strong binding affinity and providing a theoretical basis for MXene's radical scavenging capability. Using chemical reagent methods, we assessed the RNS scavenging ability (DPPH, ABTS, ONOO^−^ radicals) in Fig. 2D and E and S6 and ROS scavenging ability (PTIO, •OH, O_2_^−^ radicals) in Fig. 2F and G and S7 of QM(Zn) NPs, where the drug concentration corresponded to the Ti_3_C_2_ MXene content within the NPs. QM(Zn) NPs scavenged both RNS and ROS in a concentration-dependent manner. Specifically, at Ti_3_C_2_ MXene concentrations ≥80 μg/mL within the NPs, over 75% of ABTS, DPPH, and ONOO^−^ radicals were eliminated; similarly, ROS scavenging achieved >75% at high concentrations. These results indicate that QM(Zn) NPs possess robust scavenging capabilities for diverse ROS and RNS (Fig. 2H). Furthermore, antioxidant experiments using Ti_3_C_2_ MXene released from QM(Zn) NPs showed no significant decrease in activity compared to pristine Ti_3_C_2_ MXene at equivalent concentrations (Fig. 2I and J). To verify the protective effect of QM(Zn) NPs against oxidative stress in cells, we employed an H_2_O_2_-induced oxidative stress model in RAW 264.7 cells. Cell viability (Fig. 2K) was significantly reduced in the H_2_O_2_ group, but treatment with QM(Zn) NPs effectively prevented this damage and enhanced survival. Using DCFH-DA fluorescent dye as an intracellular ROS probe (Fig. 2L), intense green fluorescence (indicating high ROS levels) was observed in the H_2_O_2_ group, whereas the QM(Zn) NPs group exhibited minimal fluorescence, comparable to the control group. Live/dead staining results (Fig. 2M and N) further confirmed that QM(Zn) NPs effectively scavenge intracellular ROS and protect cells from oxidative damage.

### 2.3 Biocompatibility of QM (Zn) NPs

**Fig. 3:**
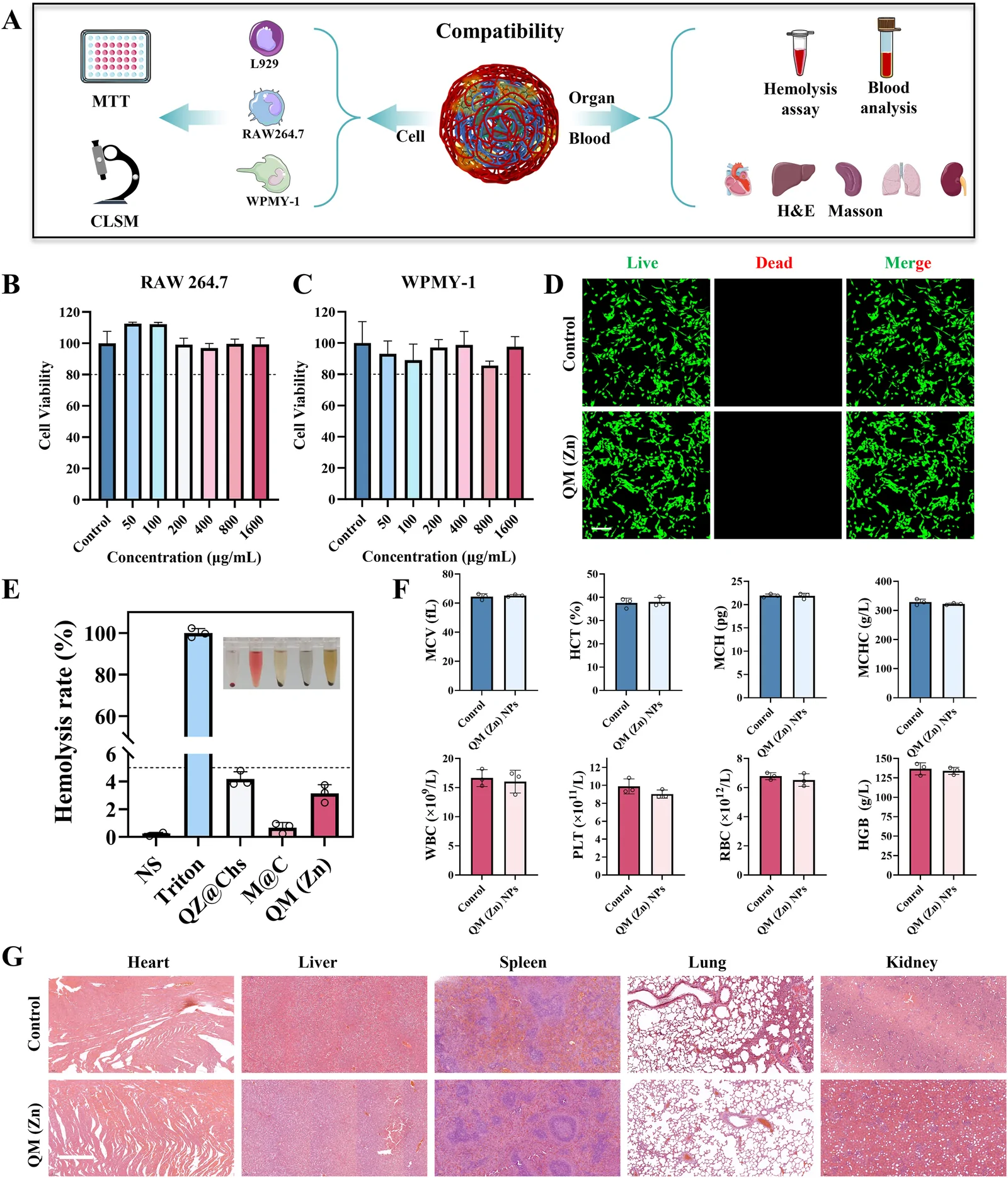
Biocompatibility of QM (Zn) NPs. **A**, The schematic illustration of cytocompatibility experiment. **B**, The cell viability of RAW 264.7 cells treated with QM (Zn) NPs. n = 3. **C**, The cell viability of WPMY-1 cells treated with QM (Zn) NPs. n = 3. D, The representative live/dead staining of WPMY-1 cells after incubation with QM (Zn) NPs. Scale bar = 200 μm. **E**, The hemolytic activity assay. n = 3. **F**, The blood analysis. n = 3. There was no significant difference between the Control group and the QM (Zn) NPs group. **G**, The representative H&E staining images of major organs of rats treated with QM (Zn) NPs. Scale bar = 400 μm. All values are expressed as mean ± SD.

Biocompatibility, a prerequisite for drug application within the body to prevent cellular damage, necessitates comprehensive evaluation (Fig. 3A). Consequently, we assessed the cytocompatibility of QM(Zn) NPs using MTT and live/dead staining assays in prostate stromal cells (WPMY-1), macrophages (RAW 264.7), and fibroblasts (L929). MTT assay results (Fig. 3B and C, S8) showed that viability of all three cell types treated with QM(Zn) NPs remained above 80% across a wide concentration range (50-1600 μg/mL), demonstrating no significant toxicity. Live/dead staining results (Fig. 3D–S9, S10) were consistent with the normal control group: cells treated with QM(Zn) NPs exhibited green fluorescence (indicating live cells) with no detectable dead cells, and cell density was comparable to the untreated group. These findings collectively indicate that QM(Zn) NPs do not induce cellular damage or toxicity.

Given the necessity of intravenous administration for QM(Zn) NPs, their blood compatibility is critically important. We therefore evaluated hemocompatibility through hemolysis testing and comprehensive blood analysis. As shown in Fig. 3E, hemolysis rates in erythrocyte suspensions remained below 5% for QZ@Chs, M@C, and QM(Zn) NPs after 3h treatment, supporting intravenous applicability. Blood analysis (Fig. 3F) revealed no statistically significant differences in red blood cells, white blood cells, or platelet counts between treatment and control groups, confirming no damage to blood components. To further assess systemic safety, we performed H&E and Masson staining on major organs of Sprague-Dawley rats following 30-day intravenous QM(Zn) NP administration. Fig. 3G and S11 demonstrate no significant physiological differences between groups: cardiomyocytes showed optimal alignment without inflammation; hepatic sinusoids displayed no hypertrophy or congestion; splenic tissues maintained normal white/red pulp distribution; lung tissues exhibited no alveolar dilatation or collapse; and renal tissues contained normal glomeruli and tubules. The histopathological analysis of major organs from the 90-day toxicity study (provided in Fig. S12) is consistent with the results mentioned above. Critically, all organs showed no pathological abnormalities, necrosis, or inflammatory infiltration, confirming QM(Zn) NPs induce negligible systemic toxicity and their metabolites cause no tissue damage under prolonged use conditions.

### 2.4 Targeting capacity and regulation of macrophages

**Fig. 4:**
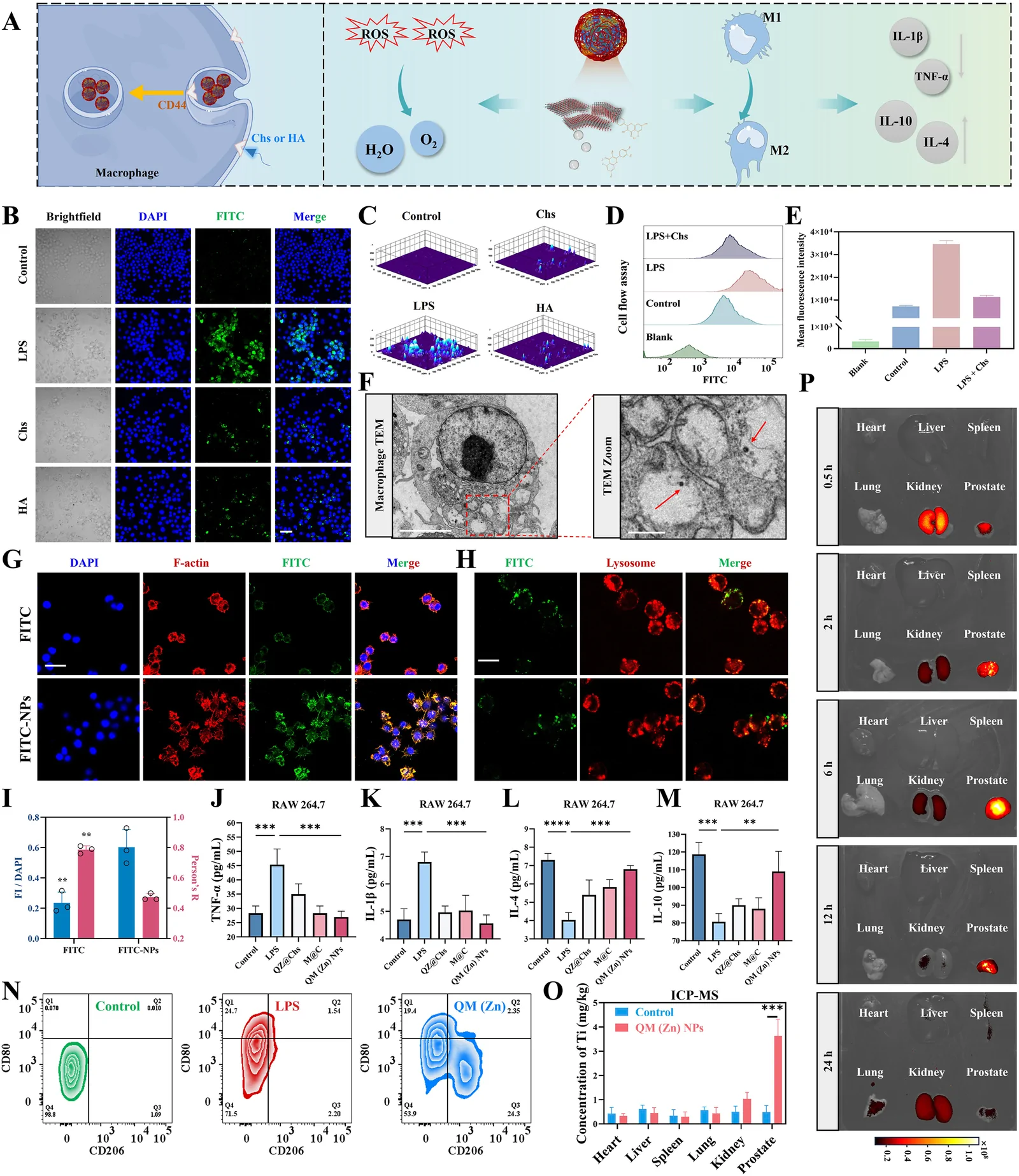
Targeting capacity and regulation of macrophages. **A**, The schematic illustration of the cellular uptake assay and regulating macrophages. **B**, The representative staining images of CD44 receptor-mediated uptake of QM (Zn) NPs by inflammatory macrophages. Scale bar = 50 μm. **C**, The fluorescence density and fluorescence distribution of stained images. **D**, The flow assay results of CD44 receptor-mediated uptake by macrophages. **E**, The statistical results of the average fluorescence density of flow experiments. n = 3. **F**, The TEM images of macrophage endocytosis. Scale bar = 5 μm and 1 μm. **G**, The comparative experiments of FITC and FITC-NPs uptake by macrophages. Scale bar = 30 μm. **H**, Lysosomal escape assay. Scale bar = 15 μm. **I**, The quantitative statistical results of uptake experiments and lysosomal escape experiments. n = 3. **J**, The TNF-α expression in macrophages. n = 3. **K**, The IL-1β expression in macrophages. n = 3. **L**, The IL-4 expression in macrophages. n = 3. **M**, The IL-10 expression in macrophages. n = 3. **N**, The flow assay for macrophage polarization. **O**, The distribution of Ti in various organs. n = 3. **P**, The representative NIR fluorescence pictures of prostatitis rats with Cy7-labeled QM (Zn) NPs at various periods. All values are expressed as mean ± SD. Statistical significance was determined using a one-way ANOVA with Tukey's multiple comparisons. **P < 0.01, ***P < 0.001, ****P < 0.0001.

The therapeutic efficacy of pharmaceutical agents is fundamentally contingent upon their capacity to achieve targeted delivery to diseased tissues. Chondroitin sulfate (Chs), a naturally occurring glycosaminoglycan, demonstrates high-specificity binding affinity for CD44 receptors transmembrane glycoproteins markedly overexpressed on activated inflammatory macrophages. This molecular recognition mechanism enables selective homing of QM(Zn) NPs to sites of inflammation (Fig. 4A). To systematically validate this targeting hypothesis, we established an in vitro inflammatory paradigm by stimulating murine-derived RAW 264.7 macrophages with bacterial lipopolysaccharide (LPS). Competitive binding inhibition assays were subsequently implemented using hyaluronic acid (HA) and Chs as molecular antagonists, pre-incubating these blockers with macrophages prior to QM(Zn) NP exposure. Fluorescence microscopic evaluation (Figures S13, 4B-4C) revealed pronounced intracellular green fluorescence signals, indicative of nanoparticle accumulation, within LPS-activated macrophages compared to quiescent controls. Critically, pre-treatment with either HA or Chs substantially diminished this fluorescence signature, confirming competitive disruption of CD44-mediated endocytic pathways. Flow cytometric quantification (Fig. 4D–E) corroborated these observations, demonstrating significantly elevated cellular uptake in inflammatory macrophages relative to baseline conditions, while blocker-treated groups exhibited markedly attenuated internalization. As illustrated in Fig. 4F, TEM analysis revealed the internalization of macrophages, and upon higher magnification, the formation of vesicular structures by the macrophages for nanoparticle endocytosis was observed. Subsequently, in Fig. 4G, a comparison was made of the difference in macrophage uptake of free FITC and FITC labeled on nanoparticles. The cytoskeleton was stained with ghost pen cyclic peptide, which demonstrated that FITC grafted on nanoparticles was taken up more by macrophages. This phenomenon was attributed to the specific recognition of macrophages by Chs in the nanoparticles, facilitating their uptake. The quantitative statistics in Fig. 4I were consistent with the staining results. The therapeutic efficacy of nanoparticle delivery is contingent upon the successful internalization of the particles by the target cells, a process that must not be captured by lysosomal. Therefore, it has been demonstrated that the nanoparticles are capable of lysosomal escape, as evidenced by the results presented in Fig. 4H. The fluorescently labeled nanoparticles (FITC) have been shown to evade the lysosomal environment, while the unbound FITC remains confined within it. The statistics of co-localization coefficients in Fig. 4I also demonstrated the same results.

To evaluate whether QM(Zn) NPs modulate macrophage functionality post-internalization, we assessed intracellular inflammatory factor expression *via* ELISA. Analysis revealed that QM(Zn) NPs significantly suppressed pro-inflammatory cytokine production while concurrently enhancing anti-inflammatory mediator expression (Fig. 4J–M). This dual regulatory effect demonstrated potent anti-inflammatory activity and suggested capacity to reprogram macrophage polarization states. Macrophage phenotypes exhibit distinct functional characteristics: classically activated M1 macrophages propagate inflammation through abundant pro-inflammatory factor secretion, whereas alternatively activated M2 macrophages promote resolution through anti-inflammatory signaling. To investigate polarization dynamics, flow cytometry was employed using CD80 as an M1-specific surface marker and CD206 as an M2-specific identifier. Under LPS stimulation, macrophages underwent rapid M1 polarization; however, QM(Zn) NP treatment profoundly attenuated this pro-inflammatory shift, instead promoting transition toward the immunoregulatory M2 phenotype (Fig. 4N–S14). Fig. S15 shows minimal polarization of M0-type RAW 264.7 macrophages towards the M2 phenotype following treatment with QM(Zn) NPs alone. To establish *in vivo* targeting efficacy, systemic distribution of intravenously administered QM(Zn) NPs was evaluated in rat models. Inductively coupled plasma mass spectrometry (ICP-MS) analysis of major organs demonstrated striking titanium enrichment specifically within prostate tissue, with only minimal accumulation in extracapsular organs including heart, liver, spleen, lungs, and kidneys (Fig. 4O). Complementary near-infrared fluorescence imaging of Cy7-labeled nanoparticles revealed intense focal fluorescence localized to the prostate region within hours post-injection, reaching peak intensity at intermediate timepoints and exhibiting sustained retention. Other organs displayed negligible fluorescence signals (Fig. 4P). Critically, comparative imaging confirmed substantially enhanced prostate accumulation of nanoparticle-conjugated Cy7 versus free dye controls (Fig. S16). Additionally, in Fig. S17, following intravenous injection, QM(Zn) NPs achieved a maximum plasma concentration (C_0_) instantaneously. The serum concentration then declined in a biphasic manner: an initial rapid distribution phase as the nanoparticles entered tissues, followed by a slower elimination phase mediated by the mononuclear phagocyte system.

These collective findings establish that QM(Zn) NPs achieve targeted delivery to inflammatory prostate microenvironments, where they orchestrate macrophage polarization from pro-inflammatory M1 toward regenerative M2 phenotypes, thereby creating an anti-inflammatory milieu conducive to tissue repair.

### 2.5 Ability to protect prostate cells

**Fig. 5:**
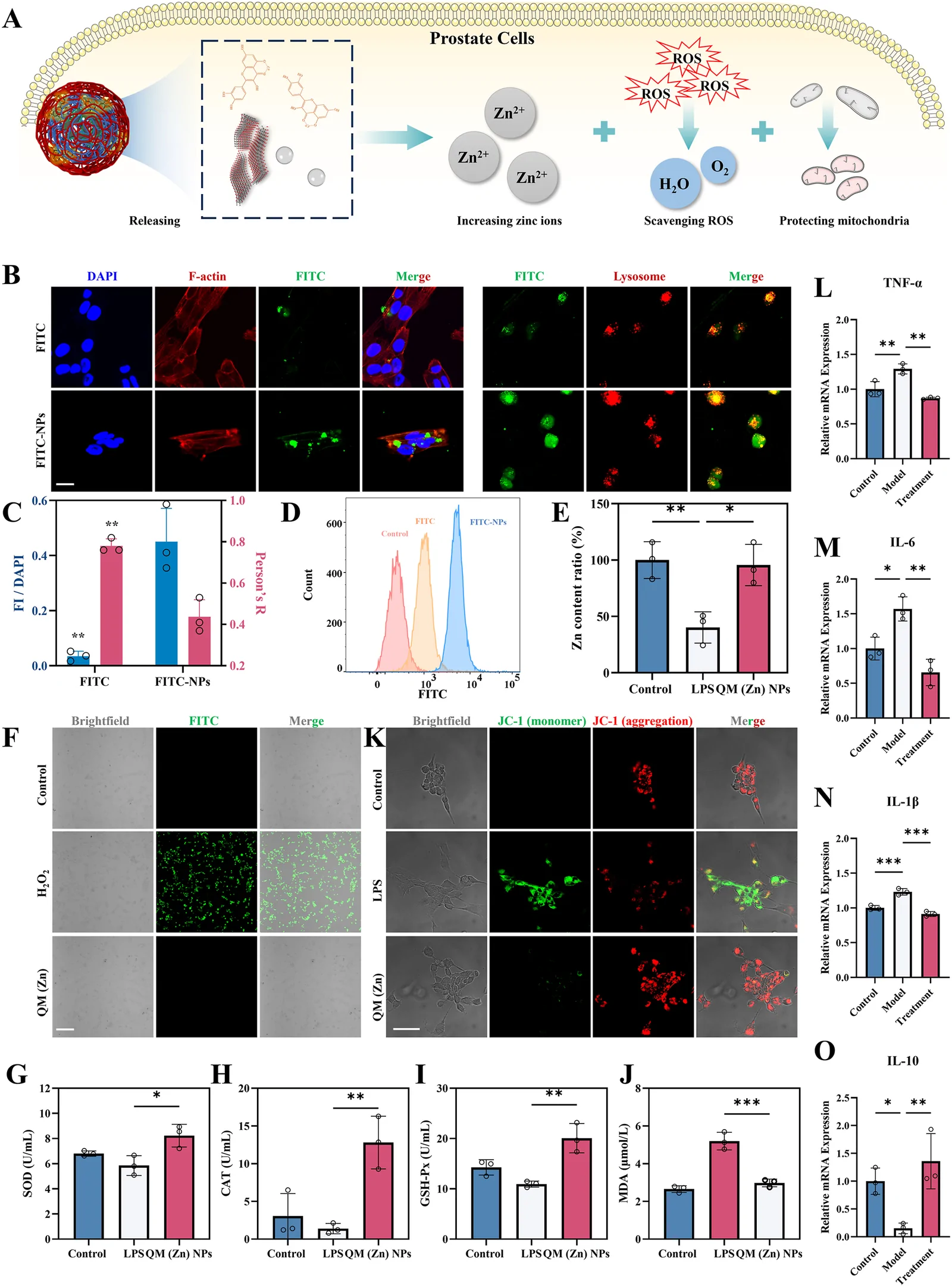
Ability to protect prostate cells. **A**, The schematic illustration of the mechanism to protect prostate cells. **B**, The cellar uptake and lysosomal escape assay. Scale bar = 20 μm. **C**, The quantitative statistical results of uptake experiments and lysosomal escape experiments. n = 3. **D**, The flow assay results of cellar uptake. **E**, The detection of intracellular zinc ion content. n = 3. **F**. The representative ROS staining images of WPMY-1 cells. Scale bar = 200 μm. **G**, The SOD expression in WPMY-1 cells. n = 3. **H**, The CAT expression in WPMY-1 cells. n = 3. **I**, The GSH-Px expression in WPMY-1 cells. n = 3. **J**, The MDA expression in WPMY-1 cells. n = 3. **K**. The representative JC-1 staining images of WPMY-1 cells. Scale bar = 60 μm. **L**, The relative mRNA expression of TNF-α in WPMY-1 cells. n = 3. **M**, The relative mRNA expression of IL-6 in WPMY-1 cells. n = 3. **N**, The relative mRNA expression of IL-1β in WPMY-1 cells. n = 3. **O**, The relative mRNA expression of IL-10 in WPMY-1 cells. n = 3. All values are expressed as mean ± SD. Statistical significance was determined using a one-way ANOVA with Tukey's multiple comparisons. *P < 0.05, **P < 0.01, ***P < 0.001.

The cytoprotective efficacy of QM(Zn) NPs against prostate inflammation was systematically investigated using prostate stromal cells (WPMY-1, Fig. 5A), beginning with comprehensive analysis of cellular internalization dynamics. As illustrated in Fig. 5B and C, the capacity of free FITC and FITC-labeled nanoparticles to be internalized by WPMY-1 and to evade lysosomes was comparatively analyzed. The results demonstrate that FITC-labeled nanoparticles are more efficiently taken up by WPMY-1 and escape from the lysosome, while free FITC is taken up in minimal amounts and is completely phagocytosed by the lysosome. These qualitative observations received rigorous quantitative validation through flow cytometric intensity profiling (Fig. 5D), confirming order-of-magnitude superior cellular internalization of the nanoparticle system. Critically, beyond delivery advantages, QM(Zn) NPs directly address the pathophysiological zinc deficiency characterizing prostatitis. When prostate cells underwent LPS-induced inflammatory perturbation, intracellular zinc reservoirs became severely depleted due to dysregulated zinc transporter expression, whereas QM(Zn) NP treatment restored physiological zinc homeostasis (Fig. 5E) through sustained release of bioavailable Zn^2+^ ions that reactivated zinc-dependent transcription factors. This metal ion delivery function was integrated with multi-tiered antioxidant protection: following hydrogen peroxide-induced oxidative challenge, QM(Zn) NPs substantially attenuated pathological ROS bursts (Fig. 5F–S18) while simultaneously upregulating endogenous defense systems. Biochemical assays demonstrated potentiated activities of metalloenzyme antioxidants - specifically superoxide dismutase (which requires zinc cofactors) and catalase, along with significant reduction in malondialdehyde (MDA), the terminal aldehyde product of membrane lipid peroxidation (Fig. 5G–J). Damage to cells results in impaired mitochondrial function within the cell and altered mitochondrial membrane potential. Poor cellular status has been demonstrated to result in a decrease or loss of mitochondrial membrane potential. Consequently, to further substantiate the protective efficacy of QM (Zn) NPs on WPMY-1 cells, the mitochondrial membrane potential was assessed utilizing a JC-1 staining kit. The outcomes are presented in Fig. 5K and S19. Subsequent to the induction of inflammation in WPMY-1 cells by LPS, a marked enhancement in green fluorescence was observed, accompanied by a concomitant weakening of red fluorescence. This finding serves as a hallmark of weakened mitochondrial membrane potential and severe cellular damage. It is evident that the mitochondrial membrane potential was restored following the treatment of QM (Zn) NPs, thereby substantiating the efficacy of QM (Zn) NPs in providing protective effects to the cells. Furthermore, the mRNA levels of inflammatory factors in WPMY-1 cells were detected by PCR experiments (Fig. 5L–O and S20), which demonstrated that QM (Zn) NPs could up-regulate anti-inflammatory factors and down-regulate pro-inflammatory factors to alleviate cellular inflammation.

### 2.6 *In Vivo* Anti-CNP effects

Prior to the therapeutic inflammation of chronic prostatitis, an acute nonbacterial prostatitis model was constructed in rats. QM (Cu) NPs, prepared in a previous study, were utilized as a control to initially assess their therapeutic efficacy (see Fig. S21). As illustrated in Fig. S21A, there was no statistically significant change in body weight among the rats during the treatment process, thereby substantiating the non-significant toxicity of the QM (Zn) NPs. As illustrated in Fig. S21B, a comprehensive examination of the external physical characteristics of the thymus and spleen was conducted for all rats within each group. It was observed that the model group exhibited prominent splenic enlargement and thymic atrophy, indicating a substantial inflammatory response. The study found that the QM (Zn) NPs group exhibited the most promising outcomes, surpassing the performance of the two intermediates group and the QM (Cu) NPs group. The prostate index results in Fig. S21C indicated that the prostate gland in the model group appeared significantly enlarged, and QM (Zn) NPs significantly alleviated this enlargement. The spleen and thymus index results, as depicted in Fig. S21D and S21E, align with the external observations presented in Fig. S21B. H&E and Masson staining of the prostate in Fig. S21F demonstrated that the prostate glandular tissue in the model group was significantly damaged, with substantial infiltration of interstitial inflammatory cells in the tissue. This finding indicates that a substantial inflammatory response had occurred. A modest recovery was observed in the QZ@Chs and M@C groups; however, the efficacy of this recovery was found to be inferior. The prostate gland exhibited enhanced protection in the QM(Zn) NPs group and the QM (Cu) NPs group, with the QM (Zn) NPs group demonstrating superiority over the QM(Cu) NPs group. In our previous study on QM(Cu) NPs for the treatment of bacterial prostatitis, we demonstrated that QM(Cu) NPs could exert therapeutic effects by modulating the NF-κB signaling pathway. Consequently, immunofluorescence staining was performed on prostate tissue sections, and the results presented in Fig. S22 demonstrate that QM (Zn) NPs can modulate the NF-κB signaling pathway, enhance anti-inflammatory factor expression, and reduce pro-inflammatory factor expression in the treatment of non-bacterial prostatitis. In sum, the present study demonstrated the considerable potential of QM (Zn) NPs in the treatment of non-bacterial prostatitis. The therapeutic effect for this condition was found to be superior to that of QM (Cu) NPs, suggesting that Zn^2+^ may play a significant role in this process. In summary, this segment of the experiment establishes the basis for subsequent evaluation of the efficacy of CNP.

**Fig. 6:**
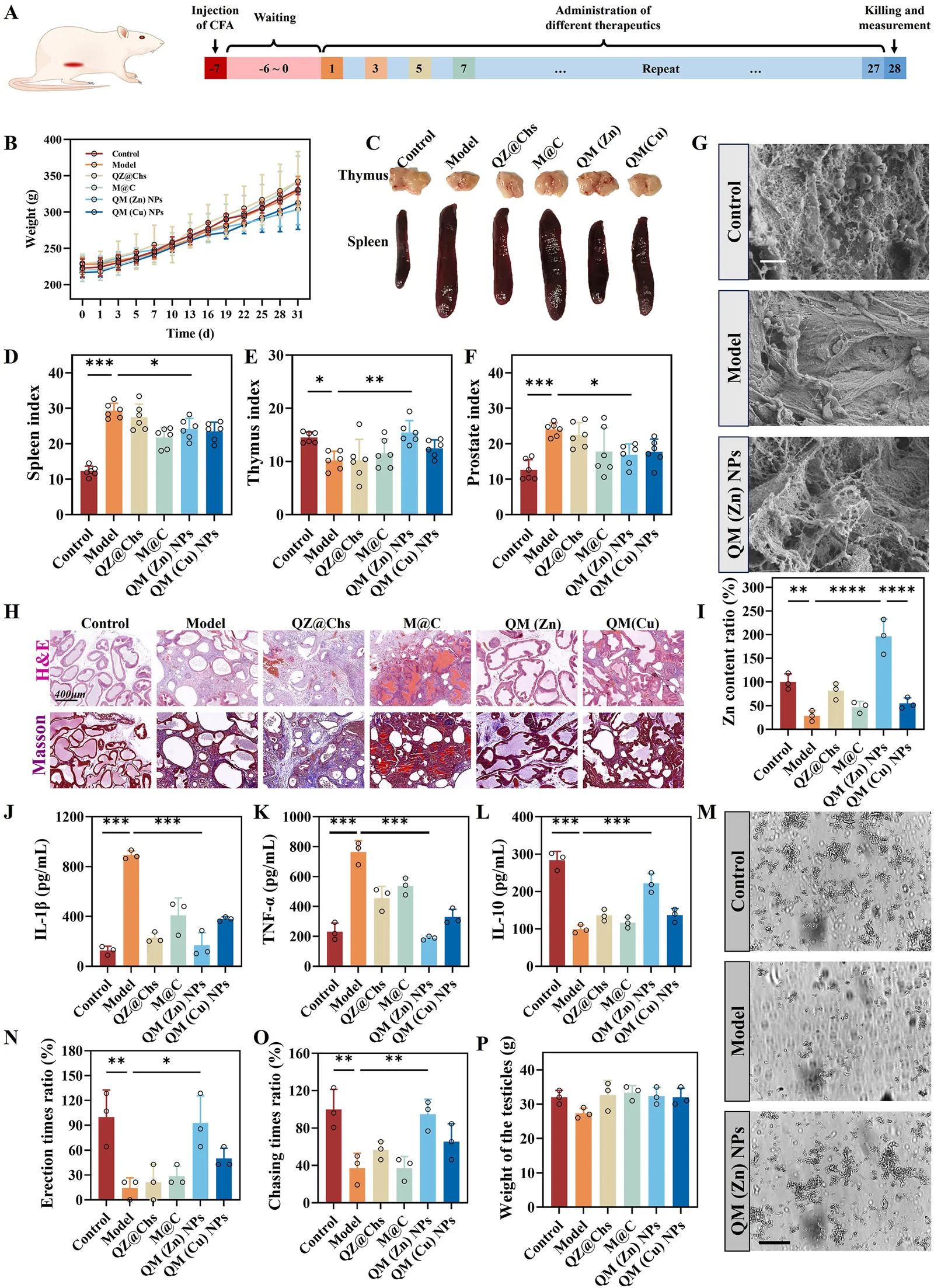
*In Vivo* anti-CNP effects of QM (Zn) NPs. **A**, The schematic illustration of animal experiment. **B**, The weight change of rats. n = 3. **C**, The representative images of thymus and spleen in rats under different treatments. **D**, The spleen indexes of rats. n = 3. **E**, The thymus indexes of rats. n = 3. **F**, The prostate indexes of rats. n = 3. **G**, The representative SEM image of the rat prostate. Scale bar = 5 μm. **H**, The representative H&E, and Masson staining images of prostates in rats under different treatments. Scale bar = 400 μm. **I**, The Zn content in prostates. n = 3. **J**, The expression of IL-1β in prostates of rats under different treatments. n = 3. **K**, The expression of TNF-α in prostates of rats under different treatments. n = 3. **L**, The expression of IL-10 in prostates of rats under different treatments. n = 3. **M**, The image of rat sperm. Scale bar = 200 μm. **N**, The erection times of rats under different treatments. n = 3. **O**, The chasing times of rats under different treatments. n = 3. **P**, The testicles weight of rats under different treatments. n = 3. Statistical significance was determined using a one-way ANOVA with Tukey's multiple comparisons. ∗P < 0.05, ∗∗P < 0.01, ∗∗∗P < 0.001, ∗∗∗∗P < 0.0001.

To evaluate the therapeutic efficacy of QM(Zn) NPs against chronic nonbacterial prostatitis (CNP), we implemented the experimental protocol outlined in Fig. 6A. The detection of a slightly acidic pH in the inflammatory prostate tissue microenvironment, as opposed to the near-neutral pH in normal conditions, serves as the foundation for the pH-responsive release mechanism of our nanoparticles (Fig. S23). Body weight monitoring (Fig. 6B) revealed progressive increases across all groups over one month, with no intergroup statistical significance. Morphological assessment of spleen and thymus (Fig. 6C) corroborated organ index quantitation (Fig. 6D and E), demonstrating marked splenomegaly and thymic atrophy in the model group attributable to inflammatory processes. These pathological alterations were significantly reversed by therapeutic intervention, with QM(Zn) NPs exhibiting optimal restorative efficacy.

Prostate morphometric analysis (Fig. 6F and S24) confirmed that QM(Zn) NPs effectively attenuated inflammation-induced prostate enlargement and hardness. Scanning electron microscopy (SEM) of fixed/dehydrated prostate tissues (Fig. 6G) revealed distinct microstructural differences: The normal group displayed a loosely organized reticular architecture, whereas the model group developed dense fibrotic configurations. This structural transition from porous to compact matrices signifies progressive fibrosis resulting from sustained inflammation. QM(Zn) NP treatment substantially restored tissue porosity. Complementary energy-dispersive X-ray spectroscopy (EDS) mapping (Fig. S25) detected severe zinc depletion in model group prostates, which was normalized to physiological levels following QM(Zn) NP administration. Histopathological examination (Fig. 6H) identified glandular structural disintegration and extensive inflammatory cell infiltration in model group prostates. These pathological features were ameliorated to near-normal status after QM(Zn) NP treatment. Inductively coupled plasma mass spectrometry (ICP-MS) quantification of prostate zinc (Fig. 6I) validated significant zinc deficiency in model animals, underscoring zinc's critical role in prostate homeostasis. QM(Zn) NP supplementation effectively reestablished physiological zinc concentrations. As illustrated in Fig. 6J–L and **S26**, the presence of each inflammatory factor and Substance P (SP) were detected by ELISA. The results demonstrated that QM (Zn) NPs could down-regulate pro-inflammatory factors and SP and up-regulate anti-inflammatory factors, thereby exerting therapeutic effects reducing the pain. Prolonged inflammation of the prostate gland is likely to have an effect on the reproductive function of rats. As illustrated in Fig. 6M, the spermatozoa of three distinct groups of rats were observed, and it was ascertained that the spermatozoa of rats in the model group exhibited significantly diminished sperm counts and viability. However, the sperm levels returned to normal after treatment with QM (Zn) NPs. As illustrated in Fig. 6N–P, the sexual function of rats treated with QM (Zn) NPs was assessed, revealing that QM (Zn) NPs exhibited the capacity to restore the impaired sexual function of rats with prostatitis.

To further explore the therapeutic potential, a comparative assessment was conducted between QM(Zn) NPs and the clinical first-line drug celecoxib. Both treatments showed no significant effect on body weight (Fig. S27A). However, QM(Zn) NPs exhibited a superior inhibitory effect on prostate enlargement (Fig. S27B and C). Furthermore, QM(Zn) NPs demonstrated a more pronounced mitigation of model-induced splenomegaly and thymic atrophy compared to celecoxib, as evidenced by the organ indices (Fig. S27B and D**-E**). And only the QM(Zn) NPs group exhibited an increase in zinc content in the prostate in Fig. S27F. Histopathological analysis (Fig. S27G) confirmed that both agents reduced inflammatory infiltration and tissue damage, with QM(Zn) NPs showing relatively better efficacy in restoring glandular architecture and ameliorating fibrosis.

The data presented herein indicate that QM (Zn) NPs possess remarkable CNP therapeutic properties and are capable of exerting multiple effects associated with zinc supplementation, including anti-inflammation and the protection of prostate tissues. Furthermore, these NPs have been observed to restore impaired reproductive function in rats subjected to long-term inflammation.

### 2.7 Analysis of treatment mechanisms

**Fig. 7:**
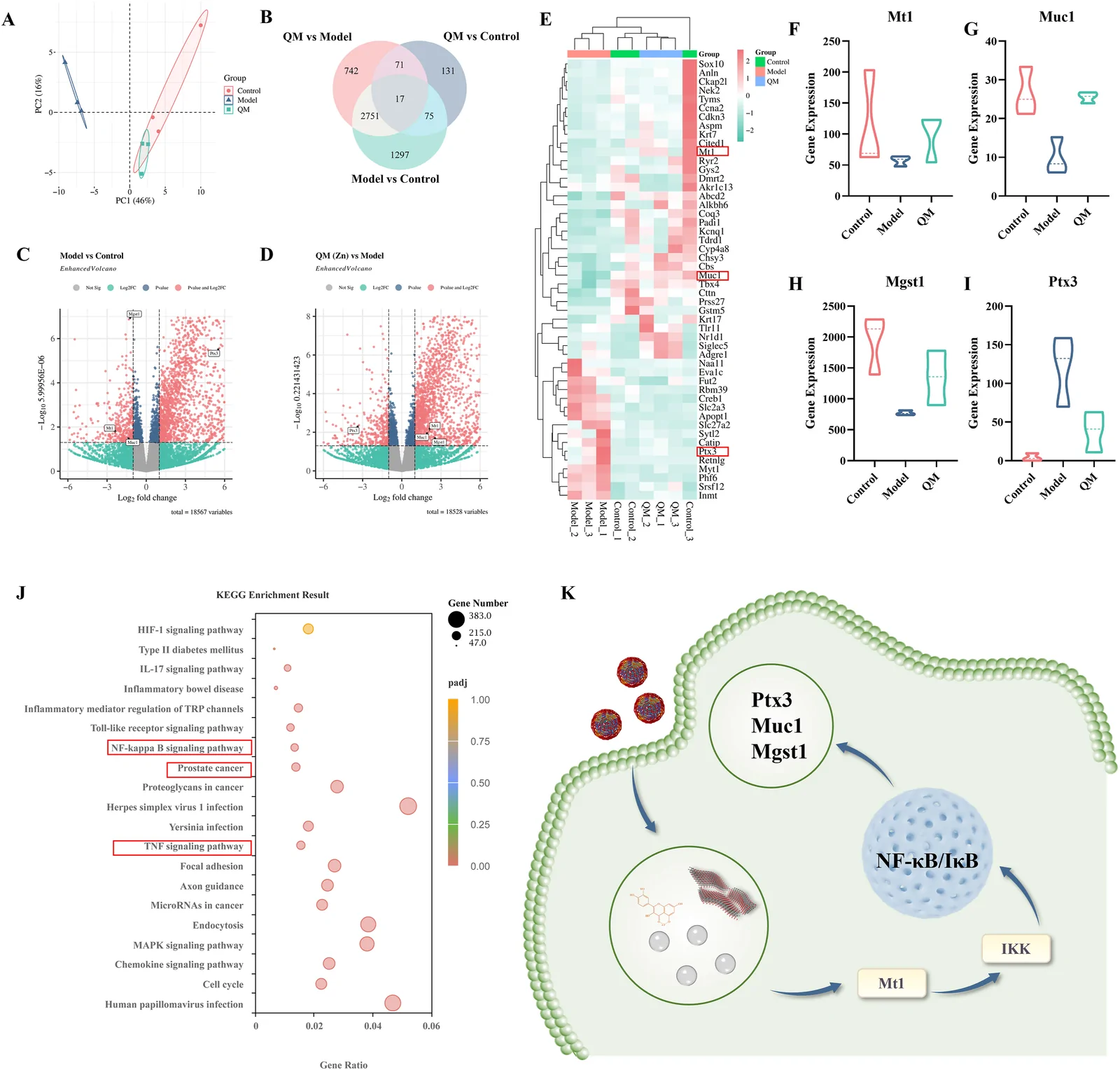
The gene expression analysis by transcriptomics (n = 3). **A**, The PCA plot of Control, Model, and QM (Zn) NPs groups. **B**, The Venn diagram of all differential genes for comparisons between groups. **C**, The volcano plot of Model and Control groups. **D**, The volcano plot of QM (Zn) NPs and Model groups. **E**, The heatmap of representative differential expressed genes in three groups. **F**, The gene expression of Mt1. n = 3. **G**, The gene expression of Muc1. n = 3. **H**, The gene expression of Mgst1. n = 3. **I**, The gene expression of Ptx3. n = 3. **J**, The KEGG pathway enrichment analysis in QM (Zn) NPs and Model groups. **K**, The schematic illustration of the mechanism.

To elucidate the potential therapeutic mechanism of QM(Zn)NPs in prostatitis, a comprehensive multi-omics analysis was conducted to characterize gene expression differences across the control, prostatitis model, and QM(Zn)NPs-treated groups. Principal component analysis (PCA) of transcriptomic data (Fig. 7A) revealed substantial disease-associated transcriptional alterations, evidenced by clear separation between control and model groups, while the QM(Zn)NPs-treated group exhibited a pronounced restorative shift toward the control profile. Differential gene expression analysis (Fig. 7B) identified significant overlap between the differentially expressed genes (DEGs) altered in the model versus control comparison and those reversed by QM(Zn)NPs treatment, suggesting targeted normalization of core disease pathways. Volcano plots (Fig. 7C–D) confirmed widespread dysregulation in the model group, with QM(Zn)NPs treatment specifically reversing expression changes in key functional genes: antioxidant genes Mt1 (metallothionein 1) and Mgst1 (microsomal glutathione S-transferase 1), significantly suppressed in the model group, were robustly upregulated post-treatment, while pro-inflammatory mediators Muc1 (mucin 1, involved in epithelial inflammation) and Ptx3 (pentraxin 3, a soluble pattern recognition receptor) showed elevated model-group expression that was effectively downregulated by QM(Zn)NPs. These patterns were corroborated by clustered heatmap analysis (Fig. 7E), which highlighted Mt1's marked specificity. As a zinc-dependent protein, the expression of metallothionein 1 is subject to regulation by the concentration of zinc ions; an increase in zinc ion concentration has been demonstrated to result in an increase in its activity [34]. Furthermore, Mt1 fulfills a variety of functions, including anti-inflammatory and antioxidant properties. Research has demonstrated that Mt1 can modulate IKK activity, thereby regulating the NF-κB/IκB pathway and exerting an anti-inflammatory effect [35]. Furthermore, Mt1, in its capacity as a highly efficient antioxidant enzyme, has been demonstrated to regulate the activity of SOD, CAT, and other enzymes, thereby assuming an antioxidant role. The gene expression data presented in Fig. 7F–I were consistent with the results obtained from the volcano and heat maps. Fig. 7J and S28 performed KEGG differential gene enrichment analysis, which revealed a higher differential gene enrichment in the NF-κB signaling pathway and inflammatory factor-related pathways. This finding is consistent with the hypothesis of the therapeutic mechanism mentioned previously. In addition to the transcriptomics data, proteomics analysis was conducted to identify differences between the groups at the protein level. The PCA result plots and Venn diagrams depicted in Fig. S29 and S30 yield conclusions that are analogous to those derived from transcriptomics. Furthermore, the volcano and heatmap results presented in Fig. S31 and S32 demonstrate the capacity of QM (Zn) NPs to modulate the expression levels of two proteins, Mmp9 and Timp1. This phenomenon may be attributed to the role of both proteins as downstream components of the NF-κB/IκB pathway. Collectively, these integrated multi-omics findings establish that QM(Zn)NPs ameliorate chronic nonbacterial prostatitis (CNP) through zinc-enhanced upregulation of MT1, which subsequently modulates the NF-κB signaling cascade *via* IKK inhibition, leading to coordinated downregulation of pro-inflammatory mediators (including Muc1, Ptx3, and MMP9), restoration of protease-antiprotease balance (MMP9/TIMP1), and activation of antioxidant defenses, ultimately resolving inflammatory and oxidative tissue damage (Fig. 7K).

**Fig. 8:**
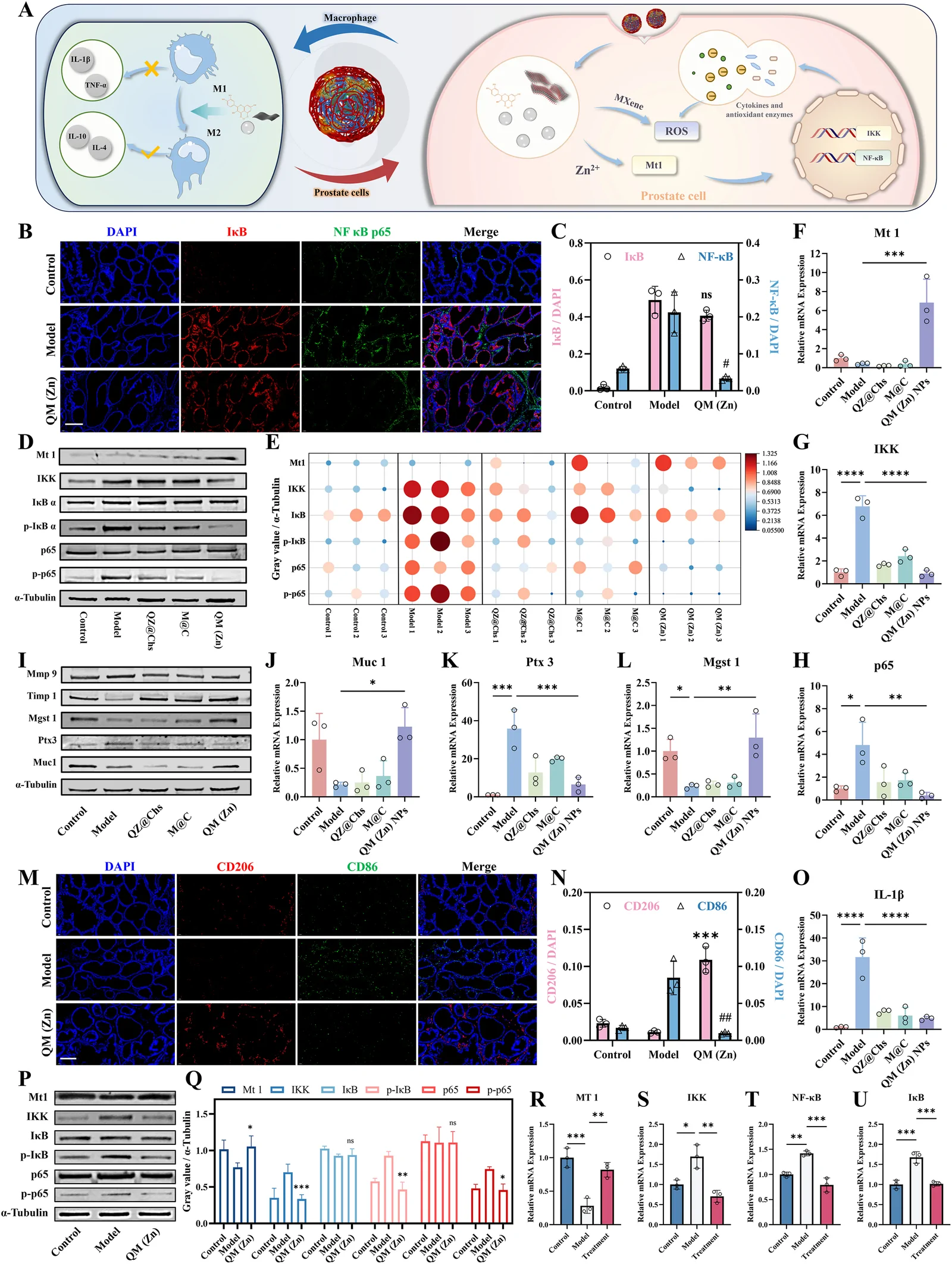
The underlying mechanism of QM (Zn) NPs in the treatment of CNP. **A**, The schematic illustration of therapeutic mechanisms. **B**, The representative fluorescent images of expression of IκB and p65 in prostates under different treatments. Scale bar = 400 μm. **C**, The fluorescence intensity statistics of IκB and p65. n = 3. ns: no significant difference, #*P* < 0.05, vs Model. **D**, The representative WB images of expression of Mt1, IKK, IκB, p-IκB, p65, and p-p65 protein in the prostate tissue. n = 3. **E**, The gray scale analysis results of WB images. n = 3. **F**, The expression of Mt1 mRNA in the prostate tissue. n = 3. **G**, The expression of IKK mRNA in the prostate tissue. n = 3. **H**, The expression of p65 mRNA in the prostate tissue. n = 3. **I**, The representative WB images of expression of Mmp9, Timp1, Mgst1, Ptx3, and Muc1 protein in the prostate tissue. n = 3. **J**, The expression of Muc1 mRNA in the prostate tissue. n = 3. **K**, The expression of Ptx3 mRNA in the prostate tissue. n = 3. **L**, The expression of Mgst1 mRNA in the prostate tissue. n = 3. **M**, The representative fluorescent images of expression of CD206 and CD86 in prostates under different treatments. Scale bar = 400 μm. **N**, The fluorescence intensity statistics of CD206 and CD86. n = 3. ***P < 0.001, ##*P* < 0.01, vs Model. **O**, The expression of IL-1β mRNA in the prostate tissue. n = 3. **P**, The representative WB images of expression of Mt1, IKK, IκB, p-IκB, p65, and p-p65 protein in the WPMY-1 cells. n = 3. **Q**, The gray scale analysis results of WB images. n = 3. **R**, The expression of Mt1 mRNA in the prostate tissue. n = 3. **S**, The expression of IKK mRNA in the prostate tissue. n = 3. **T**, The expression of NF-κB mRNA in the prostate tissue. n = 3. **U**, The expression of IκB mRNA in the prostate tissue. n = 3. All values are expressed as mean ± SD. Statistical significance was determined using a one-way ANOVA with Tukey's multiple comparisons. *P < 0.05, **P < 0.01, ***P < 0.001, ****P < 0.0001.

To validate the hypothesis based on histologic data (Fig. 8A), detailed mechanistic investigations were conducted. Immunofluorescence staining (Fig. 8B and C) showed significant upregulation of NF-κB p65 and IκB in the model group, presumably due to dissociation of NF-κB p65-IκB complexes under inflammatory conditions. By contrast, QM(Zn) NPs treatment markedly reduced protein expression. Western blot (WB) and PCR analyses further confirmed pathway involvement: as depicted in Fig. 8D and E, QM(Zn) NPs significantly upregulated Mt1 protein, which inhibited IKK. This inhibition blocked IκB and p65 phosphorylation, thereby alleviating inflammation. PCR results (Fig. 8F–H, S33) were consistent with WB findings, demonstrating that QM(Zn) NPs modulated the NF-κB pathway *via* Mt1 regulation. Immunofluorescence staining (Fig. S34–S35) evaluated downstream inflammatory factors identified by proteomics. Pro-inflammatory Mmp9 was markedly elevated in the model group but decreased in the QM(Zn) NPs group, whereas its inhibitor Timp1 showed the opposite trend. WB analysis of NF-κB downstream factors (Fig. 8I–S36) confirmed consistent Mmp9 and Timp1 expression patterns. Under oxidative stress-induced inflammation, QM(Zn) NPs upregulated Mgst1, an antioxidant protein, at the inflammatory site—findings aligning with transcriptomics data. This suggests QM(Zn) NPs exert anti-inflammatory effects by modulating the NF-κB pathway, reducing Ptx3 and increasing Muc1. PCR results (Figures S37-S38, 8J-L) validated transcriptional regulation of these proteins, consistent with WB data. To assess macrophage polarization, we stained for M1 (CD86) and M2 (CD206) markers (Fig. 8M and N). Results showed QM(Zn) NPs promoted macrophage transition from the pro-inflammatory M1 to the anti-inflammatory M2 phenotype, consistent with cellular-level experiments. PCR analysis of inflammatory cytokines (IL-1β, TNF-α, IL-4, IL-10; Fig. 8O and S39) further confirmed macrophage regulatory effects. Cellular-level validation in rat prostate tissue complemented molecular findings. WB experiments (Fig. 8P and Q) showed QM(Zn) NPs regulated Mt1 to affect IKK activity and NF-κB signaling, confirmed by PCR (Fig. 8R–U). Expression of downstream factors (Mmp9, Timp1, Mgst1) was validated *via* WB and PCR (Fig. S40–S45), matching prostate tissue results. To definitively establish the causal role of Mt1 activation in the anti-inflammatory mechanism of QM(Zn) NPs, we performed a loss-of-function experiment using siRNA-mediated gene knockdown. As shown in Fig. S46, transfection of WPMY-1 cells with Mt1-specific siRNAs led to a substantial reduction in Mt1 protein expression. Under these knockdown conditions, the inhibitory effects of QM(Zn) NPs on IKK and NF-κB p65 phosphorylation were significantly attenuated, providing direct evidence that the anti-inflammatory action of QM(Zn) NPs is dependent on Mt1 upregulation. Furthermore, we investigated whether Mt1 mediates intracellular Zn^2+^ retention. As demonstrated in Fig. S47, cells treated with QM(Zn) NPs exhibited significantly elevated intracellular Zn^2+^ content compared to untreated controls, confirming effective Zn^2+^ delivery and retention. Importantly, Mt1 knockdown prior to QM(Zn) NP treatment markedly reduced the intracellular Zn^2+^ level relative to the scramble siRNA control group, functionally validating the Zn^2+^–Mt1 interaction in our system. Collectively, these results establish Mt1 not merely as a biomarker but as a pivotal upstream mediator that couples intracellular Zn^2+^ to suppression of the IKK/NF-κB signaling axis, thereby resolving inflammation in the prostate microenvironment.

In conclusion, these experiments systematically characterize the molecular mechanism of QM(Zn) NPs in treating CNP: Mt1-mediated regulation of IKK activity modulates the NF-κB pathway, thereby regulating downstream inflammatory and oxidative stress response factors. These findings provide robust evidence for QM(Zn) NPs as a potential therapeutic for CNP.

### 2.8 Ability to relieve pain in CNP rats

**Fig. 9:**
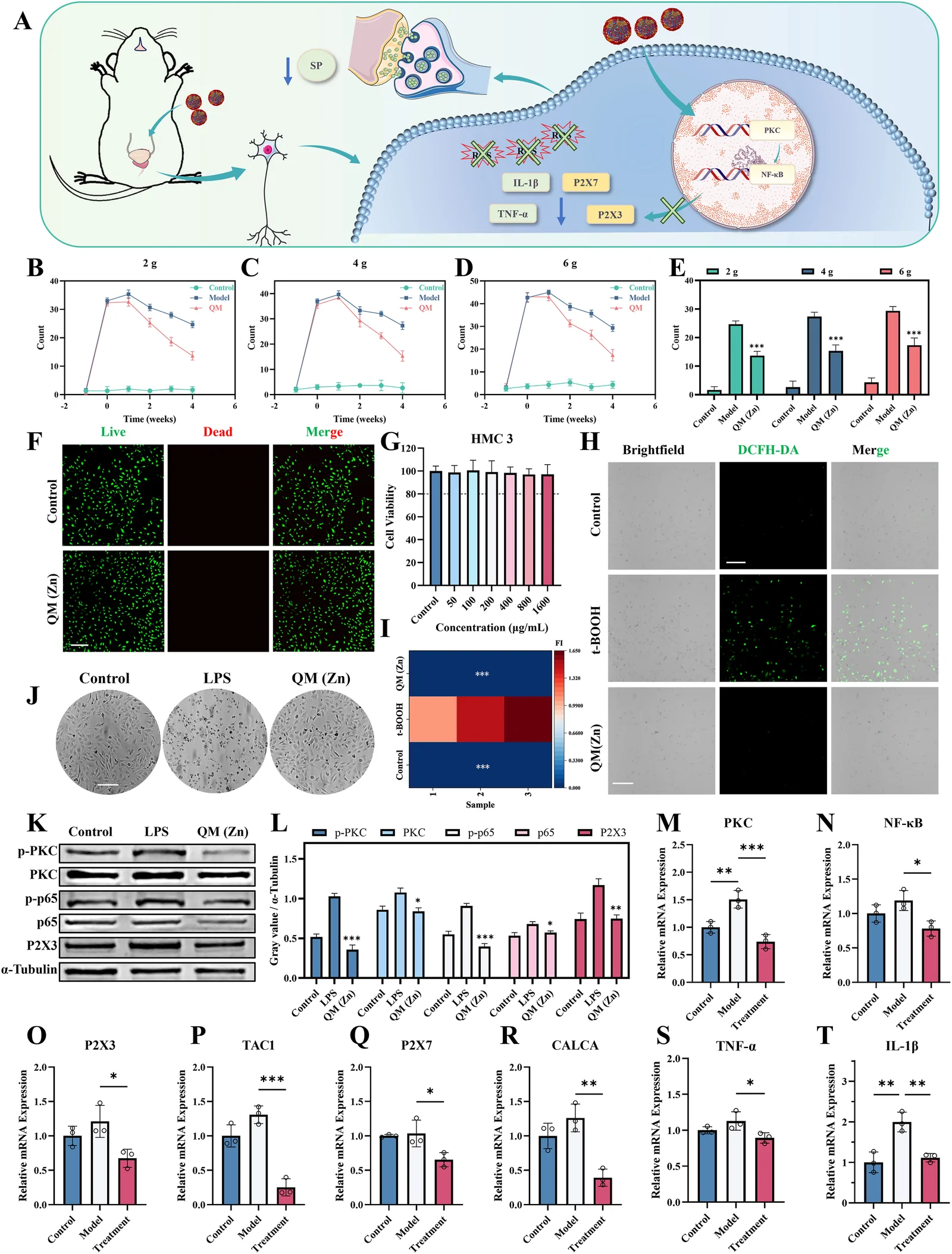
The ability to relieve pain in CNP rats. **A**, The schematic illustration of relieving pain mechanisms. **B**, The nociceptive test data using 2 g fiber filaments. n = 3. **C**, The nociceptive test data using 4 g fiber filaments. n = 3. **D**, The nociceptive test data using 6 g fiber filaments. n = 3. **E**, The statistical results of the fourth week of pain testing. n = 3. **F**, The representative live/dead staining of HMC3 cells after incubation with QM (Zn) NPs. Scale bar = 200 μm. **G**, The cell viability of HMC3 cells treated with QM (Zn) NPs. n = 3. **H**, The representative ROS staining images of HMC3 under t-BOOH and QM (Zn) NPs treatment. Scale bar = 200 μm. **I**, The fluorescence semi-quantitative statistical results. n = 3. **J**, The representative images of HMC3 under LPS and QM (Zn) NPs treatment. Scale bar = 100 μm. **K**, The representative WB images of expression of p-PKC, PKC, p-p65, p65, and P2X3 protein in the HMC3 cells. n = 3. **L**, The gray scale analysis results of WB images. n = 3. **M**, The expression of PKC mRNA in the prostate tissue. n = 3. **N**, The expression of NF-κB mRNA in the prostate tissue. n = 3. **O**, The expression of P2X3 mRNA in the prostate tissue. n = 3. **P**, The expression of TAC1 mRNA in the prostate tissue. n = 3. **Q**, The expression of P2X7 mRNA in the prostate tissue. n = 3. **R**, The expression of CALCA mRNA in the prostate tissue. n = 3. **S**, The expression of TNF-α mRNA in the prostate tissue. n = 3. **T**, The expression of IL-1β mRNA in the prostate tissue. n = 3. All values are expressed as mean ± SD. Statistical significance was determined using a one-way ANOVA with Tukey's multiple comparisons. *P < 0.05, **P < 0.01, ***P < 0.001.

Long-term pain is one of the prominent symptoms of chronic prostatitis, which seriously affects the quality of life of patients, and pain relief is an important goal in the treatment of CNP. Therefore, we examined the changes in pain perception in each group of rats by the Von Frey Fiber Wire Nociceptor. Briefly, rats were placed in a wire mesh cage, waited for them to calm down, and then stimulated the lower abdomen and scrotum of the rats with different strengths of the fiber wire, and the rats' movement of the test site away from the fiber wire was recorded as a pain response, which was examined weekly, and each strength of the fiber wire was tested 50 times each. The results are displayed in Fig. 9B–D. The rats in the control group demonstrated minimal response to fiber filaments of 2g to 6g strengths, indicating a high pain threshold. The rats in the model and treatment groups exhibited minimal response to the three types of fiber filaments one week prior to modeling. In contrast, rats after modeled demonstrated a response rate of over 30 times for the 50 tests of 2g fiber filaments and more than 45 times for the 50 tests of 6g fiber filaments. These findings indicate a reduction in the pain threshold and an enhancement in sensitivity to pain in chronic non-bacterial prostatitis rats. Over the course of four weeks of treatment, the number of responses in the model group demonstrated a gradual decline, likely attributable to the rats' intrinsic self-healing capacity. Conversely, the rats in the treatment group exhibited a substantial decrease in their pain response, with a rate of decrease that was significantly faster than that observed in the model group. In the statistical species of the number of responses in the fourth week of Fig. 9E, the treatment group exhibited a response frequency of approximately 10, and its sensitivity to pain was significantly lower than that of the model group. The experimental results suggest that the CNP rats treated with QM (Zn) NPs exhibit not only a mitigated inflammatory response but also a substantial alleviation of pain.

In order to elucidate the potential mechanism by which QM (Zn) NPs could alleviate pain in CNP rats, a preliminary exploration was conducted using human-derived microglia HMC 3. First, in Fig. 9F and G, we verified that QM (Zn) NPs were not obviously toxic to HMC 3 cells by live-dead fluorescence staining and MTT experiments. The treated groups showed green fluorescence, no dead cells existed, and cell viability was good in all groups, which was above 80%. Subsequently, the protective ability of QM (Zn) NPs against HMC 3 cells was verified. The oxidative stress of the cells was modeled through the application of tert-butyl hydroperoxide (t-BOOH), a stimulant that induced the production of reactive oxygen species, thereby inflicting damage upon the cells. As demonstrated in Fig. 9H and I, considerable green fluorescence was observed in the cells treated with t-BOOH following DCFH-DA staining, indicative of the generation of reactive oxygen species. The application of QM (Zn) NPs led to a substantial reduction in green fluorescence, suggesting their capacity to mitigate oxidative stress and safeguard neuronal cells. In Fig. 9J, we once more employed LPS treatment to induce the inflammation model of neuronal cells. The results demonstrated that the long spindle cell structure disappeared and the cell morphology was disrupted after LPS treatment. However, the cell morphology was restored to normal after treatment with QM (Zn) NPs. Protein and RNA from HMC3 cells were extracted for Western blot (WB) and polymerase chain reaction (PCR) experiments to preliminarily explore the mechanism by which QM (Zn) NPs protect neuronal cells. The WB results of Fig. 9K and L demonstrated that QM (Zn) NPs may regulate the expression of downstream proteins such as P2X3 by affecting the phosphorylation of PKC, which in turn affects the phosphorylation of p65. The results of the PCR analysis depicted in Fig. 9M–T demonstrate a consistent pattern, indicating that QM (Zn) NPs modulate the expression of downstream neuropathic pain-related genes, including P2X3, P2X7, and TAC1 (encoding Substance P), while concomitantly reducing the expression of inflammatory factors such as TNF-α and IL-1β, subsequent to the modulation of the PKC-p65 axis. The aforementioned results demonstrated that QM (Zn) NPs may reduce the expression of pain and inflammatory factors by regulating the PKC-p65 axis. This, in turn, protects nerve cells and achieves pain relief. Collectively, these findings demonstrate that QM(Zn) NPs exert dual neuroprotective effects: oxidative stress mitigation *via* ROS scavenging and inflammatory suppression through PKC-NF-κB p65 axis modulation. By reducing expression of pain-associated receptors (P2X3/P2X7) and pro-inflammatory mediators (TNF-α/IL-1β), QM(Zn) NPs block nociceptive signaling pathways in microglia, providing a mechanistic link to *in vivo* pain relief observed in CNP rats. These results highlight QM(Zn) NPs as a promising therapeutic for managing inflammatory pain *via* multi-targeted modulation of glial cell dysfunction. To further investigate the direct neuroprotective effect of QM(Zn) NPs, we examined their efficacy against oxidative and inflammatory injury in SH-SY5Y cells. As shown in Fig. S48, the QM(Zn) NPs exhibited no significant cytotoxicity and effectively protected cells from t-BOOH-induced excessive ROS production. Furthermore, the treatment significantly downregulated the expression of key pain and inflammatory mediators, including Substance P and pro-inflammatory cytokines.

## 3 Conclusion

Collectively, we have engineered QM(Zn)NPs, a Ti_3_C_2_ MXene-zinc composite nanoparticle system, to holistically address chronic nonbacterial prostatitis (CNP) through clinically oriented multifunctional therapy. This nanoplatform achieves targeted accumulation at inflamed prostate sites, where controlled release of Ti_3_C_2_ MXene, quercetin, and Zn^2+^ ions initiates synergistic therapeutic cascades: Ti_3_C_2_ MXene directly scavenges reactive oxygen species *via* electron-deficient surface reactions; quercetin simultaneously polarizes macrophages toward M2 phenotypes and enhances cellular zinc uptake; intracellular zinc subsequently upregulates Mt1 expression, thereby suppressing IKK/NF-κB signaling to resolve inflammation. Concomitantly, the antioxidant capacity extends to sensory neurons, providing neuroprotective analgesia that alleviates CNP-associated pain. Comprehensive biosafety assessment confirms system biocompatibility. By integrating precision targeting, multimodal anti-inflammatory action, oxidative stress mitigation, and neural protection within a unified architecture, QM(Zn)NPs establish a novel therapeutic paradigm for CNP management.

## 4 Materials and methods

### 4.1 Materials

Chondroitin sulfate (Chs), quercetin (Que), zinc (II) chloride (ZnCl_2_), N-(3-dimethylaminopropyl)-N′-ethylcarbodiimide hydrochloride (EDC⸱HCl) and levofloxacin were provided by Shanghai Macklin Biochemical Co., Ltd. 4-Dimethylaminopyridine (DMAP) was purchased from Tianjin Heowns Biochemical Co., Ltd. Carboxylated chitosan (CCS) was obtained from Shanghai Aladdin Biochemical Technology Co., Ltd. Small diameter Ti_3_C_2_Tx (MXene) thin layer dispersion was obtained from Nanjing XFNANO Materials Tech Co., Ltd.

### 4.2 Preparation of QM (Zn) nanoparticles

The synthesis of the QZ@Chs followed a methodical approach, employing a series of steps that align with our prior studies [31]. In summary, Quercetin (0.15 g) and anhydrous zinc chloride (0.036 g) were dissolved in anhydrous ethanol separately. And zinc chloride solution was added to the quercetin solution. The pH of the reaction system was then adjusted to 8 with NaOH solution (2 mol/L). A water bath at 60 °C was used to heat and stir the reaction system for 8 h. After filtration, washing and drying, Que-Zn was obtained. Chs (100 mg) was dissolved in 10 mL water and activated by EDC (110 mg) and DMAP (30 mg). Que-Zn (40 mg) was dissolved in 30 mL DMSO and added in Chs solution. After 6 h of reaction and dialysis for three days, the insoluble solids were extracted by filtration, and the solution was subsequently freeze-dried.

MXene was added to the carboxylated chitosan solution (10 mg/mL, 10 mL), and the mixture was ultrasonically stirred to combine the two and form the product M@C. The QZ@Chs (100 mg) compound was dissolved in water and subsequently added to the M@C solution. The mixture was then stirred to facilitate a reaction, resulting in the formation of QM (Zn)NPs.

### 4.3 Characterizations of QM (Zn) nanoparticles

Samples of QM (Zn) NPs solution were deposited onto a carbon film, allowed to dry, and then examined under a transmission electron microscope (TEM) (Talos L120C G2, Thermo Fisher). The morphology of the nanoparticles was then observed. The examination of the particle size, polydispersity index (PDI), and zeta potential values was conducted using a Malvern particle size analyzer. A quantity of powder samples was meticulously amalgamated with potassium bromide (KBr) powder, subsequently compressed into the form of tablets, and then subjected to rigorous analysis by means of a Fourier transform infrared (FTIR) spectrometer (Nicolet iS50, Thermo Fisher) within the spectral range of 400 to 4000 cm^−1^.The 1H NMR analysis was performed using a 400 MHz digital NMR spectrometer (JNM-ECZ400 S/L1, JEOL) with deuterium reagent as the solvent and tetramethylsilane (TMS) as the reference standard at room temperature. X-ray photoelectron spectroscopy (XPS) (ESCALAB Xi+, Thermo Fisher) was utilized to ascertain the full and narrow spectra of elements present in the nanoparticles.The sedimentation rates of MXene and QM (Zn) NPs suspended in water, phosphate-buffered saline (PBS), and serum-free cell culture medium, Dulbecco's modified Eagle medium (DMEM), were also recorded, respectively. The alterations in the dimensions of the nanoparticles were meticulously documented on days 0, 7, and 14. The ultraviolet-visible (UV-Vis) spectra of Que, Que-Zn, and MXene were scanned in the 200-800 nm wavelength range (Lambda950, PerkinElmer). The release of Que-Zn and MXene from QM(Zn)NPs was detected by placing them in acidic and neutral release media. The quantification of Que-Zn's absorbances in the release media was conducted through the utilization of UV spectroscopy, a method employed to ascertain the release profile. The release of MXene was observed using TEM.

### 4.4 Molecular dynamics simulation

Structures of all compounds were retrieved from the PubChem database [36]. REPEAT charge of Ti_3_C_2_ MXene was calculated with CP2K software [37]. Topology files for 10*10*1 supercell of TiC and CCS with 10 monomers were generated using Sobtop software, Multiwfn software and the ORCA 6.0.1 software [[38], [39], [40], [41], [42], [43], [44], [45], [46], [47], [48], [49], [50], [51]]. A cubic box with 3.08, 5.34, 9.69 nm sides was constructed containing supercell, CCS molecules and 4600 solvent molecules. Molecular dynamics (MD) simulations were performed with Gromacs software using the General AMBER Force Field (GAFF), Universal Force Field (UFF) and OPLS-AA force field (oplsaa.ff). The simulation was run for 200 ns. [52].

### 4.5 ROS/RNS-Scavenging Activities of QM (Zn) NPs

To ascertain the capacity of the solutions to scavenge free radicals, a series of nanoparticle solutions with varying concentrations were prepared.

Salicylic acid ethanol solution, ferrous sulfate solution, and hydrogen peroxide solution were prepared. Subsequently, the ferrous sulfate solution, salicylic acid solution, ultrapure water, and an appropriate amount of sample were thoroughly mixed. Thereafter, hydrogen peroxide was added. The water bath was heated for a total incubation period of 15 min, after which the absorptivity value was measured at 510 nm. The objective of this study is to assess the -OH scavenging ability.

Tris-HCl buffer was added to the samples, which were then reacted with a catechol solution for a designated period of time. The absorbance was subsequently measured at 320 nm in order to evaluate the scavenging ability of superoxide anion radicals.

DPPH was dissolved in ethanol to prepare a solution of appropriate concentration, then an equal volume of DPPH solution was mixed with the sample solution and the reaction was carried out at 37 °C for 30 min. Absorbance was measured at 517 nm to assess the scavenging ability of reactive nitrogen radicals.

The ABTS solution and potassium persulfate solution were prepared by mixing the two solutions in equal volumes and then leaving them in a cool place for 12 h to obtain the working solution. The working solution was diluted and reacted with the sample solution for 30 min, after which the absorbance was measured at 734 nm to assess the scavenging ability of reactive nitrogen radicals.

RAW264.7 cells were inoculated in confocal dishes at a density of 2 × 10^5^ cells/dish. Following a 24-h incubation period, the medium was substituted with a hydrogen peroxide-containing medium and further incubated for 12 h. The hydrogen peroxide medium was substituted with the drug medium, and cells in primary DMEM were utilized as the control. Following an additional 4-h period, the medium containing the drug was disposed of, and the cells were subjected to staining with a DCFH-DA staining kit. Subsequently, the cells were observed under a fluorescence microscope (FV3000, Olympus).

### 4.6 Biocompatibility of QM (Zn) NPs

The biocompatibility of QM (Zn) NPs was evaluated in terms of cytocompatibility and hemocompatibility. L929, RAW264.7, and WPMY-1 cells were initially inoculated in 96-well plates at a density of 1 × 10^4^ cells/well. Following a 24-h incubation period, the DMEM medium was substituted with a drug medium comprising QM (Zn) NPs. In the absence of medium replacement, cells were utilized as a control group. Subsequent to an interval of 24 h, the medium is substituted with MTT solution and incubated at 37 °C for 4 h. Subsequently, the MTT solution was disposed of, and dimethyl sulfoxide (DMSO) was added. The mixture was then incubated for 15 min. Following this, the absorbances were measured at 492 nm using an enzyme meter. Furthermore, L929, RAW264.7, and WPMY-1 cells were exposed to the highest concentration of the drug and subsequently stained with a Calcein-AM/PI live/dead cell fluorescence staining kit. The stained cells were then observed under an inverted fluorescence microscope (FV3000, Olympus).

The hemocompatibility of the QM (Zn) NPs solution was evaluated using rat erythrocytes. An equal volume of erythrocyte suspension was mixed with QM (Zn) NPs solution and incubated at 37 °C for 3 h. Following centrifugation at 3000 rpm for 10 min, the concentration of hemoglobin in the superior phase was determined by measuring the corresponding optical density at 540 nm using an enzyme marker. Furthermore, the rats were injected with QM (Zn) NPs solution, and the blood was collected and analyzed by a blood biochemistry analyzer to observe the changes of various biochemical indicators. Concurrently, the hearts, livers, spleens, lungs, and kidneys of the rats were extracted and subjected to pathological sectioning to discern any alterations in the organs.

### 4.7 Targeting Capacity of QM (Zn) NPs

Fluorescein isotope of isothiocyanate (FITC) was utilized as a probe to label QM (Zn) NPs. RAW264.7 cells were inoculated in confocal dishes at a density of 2 × 10^5^ cells/dish and cultured in lipopolysaccharide (LPS)-containing medium for 12 h to polarize them into inflammatory macrophages with high expression of CD44 receptors on their surface. Following the removal of the LPS-containing medium, the drug-containing medium was introduced, and after an incubation period of 24 h, the cells were fixed with paraformaldehyde and the nuclei were stained with 4′,6-diamidino-2-phenylindole (DAPI). The cellular uptake of the fluorescently labeled compound was observed under a fluorescence microscope. The *in vivo* targeting ability of the compound was characterized by conducting *in vivo* imaging experiments and mass spectrometry. A rat prostate model was constructed, after which the heart, liver, spleen, lung, kidney, and prostate were collected. The collection process was initiated by injecting Sulfo-Cyanine7 (Cy7)-labeled QM (Zn) NPs solution into the rat. The enrichment of nanoparticles in the prostate was verified by determining the drug distribution through fluorescence imaging and detecting the content of Ti elements in each tissue through ICP-MS.

### 4.8 Animals

Male Sprague-Dawley (SD, 200 ± 20 g) were sourced from the Laboratory Animal Center of Xi'an Jiaotong University. The rats were housed under the controlled conditions: humidity of 50–60%, temperature of 20 °C, and 12-h day and night cycles, in an IVC class animal facility (License no. SCXK (Shaanxi) 2015–002). All animal experiments complied with the ARRIVE guidelines and were carried out in accordance with the Animals (Scientific Procedures) Act 1986.

### 4.9 Establishment of the rat CNP model

Following the administration of isoflurane anesthesia, the skin of the lower abdomen of Sprague-Dawley rats was sterilized with alcohol and then incised for approximately 1 cm. Subsequently, the bladder was meticulously lifted with a sterile swab, exposing the prostate. A sterile 1-mL syringe was utilized to administer 50 μL of complete F-adjuvant into each prostate lobe. The prostate was then reset intraperitoneally with a sterile cotton swab, and the muscle and skin tissues were meticulously sutured layer by layer. Following the surgical procedure, a small quantity of penicillin was injected subcutaneously in the vicinity of the suture line, and iodine was applied to disinfect the wound. Aseptic technique is employed throughout the procedure to minimize the possibility of contamination.

### 4.10 Evaluation of the efficacy of nanoparticles

The experiment was divided into the following groups: the normal group, the model group, the QZ@Chs group (50 mg/kg), the M@C group (50 mg/kg), the QM (Zn) NPs group (50 mg/kg), and the QM (Cu) NPs group (50 mg/kg). The following groups are to be considered: the model group, the QZ@Chs group, the M@C group, the QM (Zn) NPs group, and the QM (Cu) NPs group. The prostatitis model was established on day −7. Starting from day 1, the administration of QZ@Chs, M@C, QM (Cu) NPs, and QM (Zn) NPs was executed at two-day intervals. On the thirtieth day, the rats were euthanized, and the prostate tissues and associated immune organs were harvested to assess the therapeutic effect. The body weight changes of the rats in each group were meticulously recorded, and the resulting data were systematically plotted and analyzed. The rats were euthanized on day 30 of the experiment. Subsequently, a thorough examination of the lower abdomen of the rats was conducted, and the prostate gland, situated below the bladder, was meticulously dissected and documented through photography for comparative analysis. Subsequently, the prostate was extracted and its weight was documented. Subsequently, the prostate index was calculated, and the pathological sections were stained. Concurrently, the thymus and spleen were extracted, and the weight of each was recorded to determine the thymus and spleen indices.

$$\text{Organ}\;\text{index}=\frac{tissue\;index}{body\;index}\times 10000$$

### 4.11 Enzyme-linked immunosorbent assay (ELISA)

Prostate tissue samples were collected 30 days after surgery. The enzyme-linked immunosorbent assay (ELISA) kit was utilized to quantitatively assess the expression levels of various cytokines in rat tissues.

### 4.12 Quantitative real-time PCR

The quantification of mRNA expression levels in rat prostate tissue was determined by means of quantitative real-time PCR. Rats were euthanized on day 30 after surgery. Subsequently, 80–100 mg of prostate tissue was obtained. Total RNA was extracted by grinding with Trizol reagent and adjusted to a uniform concentration after reverse transcription of the RNA into first-strand cDNA using a reverse transcription kit. The quantity of cDNA for various cytokines was then quantified using a real-time fluorescence quantitative PCR instrument.

In the course of PCR experiments conducted on prostate tissue, the sequences of primers for GAPDH were 5′-AAGTTCAACGGCACAGTCAAGG-3′ and 5′-GACATACTCAGCACCAGCATCAC-3′; MMP 9 were 5′- CAAACCCTGCGTATTTCCATTCATC-3′ and 5′- GATAACCATCCGAGCGACCTTTAG-3′; TIMP 1 were 5′- ATGGAGAGCCTCTGTGGATATGTC-3′ and 5′- ATGCCAGGGAACCAGGAAGC-3′; MGST 1 were 5′-AAACATCGTTCCCTTTCTCGGTATC-3′ and 5′-AGTCAAGTAAGCAATGGTGTGGTAG-3′; PTX 3 were 5′-TGTGATTCTGCTTTGTGCTCTCTG-3′ and 5′-GGTCCTCGGTGGGATGAAGTC-3′; MUC 1 were 5′-GTGGCTTTGGTCATCGTCTATCTC-3′ and 5′-GTCCGTGAGTGTGGTAGGTAGG-3′; MT 1 were 5′-AAGAACTGCAAATGCACCTCCTG-3′ and 5′-CACTTGTCCGAGGCACCTTTG-3′; IκB were 5′-AGGACGAGGATTACGAGCAGATG-3′ and 5′-TCTCTTCATGGATGATTGCCAAGTG-3′; NF-κB p65 were 5′-ACTGGGTGACATCTGCTTCTCC-3′ and 5′-CATCTTCTTCAGGTTCTTGGCTTCC-3′; IL-10 were 5′-GACGTGGAGCAGGTGAAGAATG-3′ and 5′-ACGTAGGCTTCTATGCAGTTGATG-3′; IL-4 were 5′-CAACAAGGAACACCACGGAGAAC-3′ and 5′-CTTCAAGCACGGAGGTACATCAC-3′; IL-1β were 5′-CCCAAACAATACCCAAAGAAGAAGATG-3′ and 5′-CTGCTTGAGAGGTGCTGATGTAC-3′; TNF-α were 5′-AGATGTGGAACTGGCAGAGGAG-3′ and 5′-TCAGTAGACAGAAGAGCGTGGTG-3′;

In the course of PCR experiments conducted on cells, the sequences of primers for GAPDH were 5′-GTGGACCTGACCTGCCGTCTAG-3′ and 5′- GAGTGGGTGTCGCTGTTGAAGTC-3′; IKK were 5′-TGGACGACCTAGAGGAGCAAGC-3′ and 5′-AAGCAGCAGCCGTACCATTTCC-3′; IκB were 5′-CCCGCACCTCCACTCCATCC-3′ and 5′-AGCATTGACATCAGCACCCAAGG-3′; NF-κB p65 were 5′-ATGGTGGTCGGCTTCGCAAAC-3′ and 5′-CGCCTCTGTCATTCGTGCTTCC-3′; MUC 1 were 5′-CTTTCTTCCTGCTGCTGCTCCTC-3′ and 5′-TTCTCTGGGTAGCCGAAGTCTCC-3′; MGST 1 were 5′-GTACGCAGAGCCCACCTGAATG-3′ and 5′-GGGGTCGGGACCACTCAAGG-3′; PTX 3 were 5′-GGCGGCTACCACTGTTGAGATG-3′ and 5′-CCACCCACACAGCAGCCATTC-3′; MMP 9 were 5′-CCCTGGTCCTGGTGCTCCTG-3′ and 5′-CTGCCTGTCGGTGAGATTGGTTC-3′; TIMP 1 were 5′-TGGCTTCTGGCATCCTGTTGTTG-3′ and 5′-CCTGATGACGAGGTCGGAATTGC-3′; PKC were 5′-GAGGAATGGAAGGGTTGGCAAGG-3′ and 5′-CACTGTGGGAGAGGAGAGGGAAG-3′; P2X3 were 5′-TGAGTACCGCACCCTCCTGAAG-3′ and 5′-GCCGCCACAGAGCTGATGATG-3′; P2X7 were 5′-TCAGGGCGGAATAATGGGCATTG-3′ and 5′-AAGGCGACGGAAACTGTATTTGGG-3′; CALCA were 5′-GCCTCCATGCAGCACCATTCAG-3′ and 5′-CTGGCCTTCATCTGCACATAGTCC-3′; TAC 1 were 5′-TCCTCGTGGCCTTGGCAGTC-3′ and 5′-TCCTCCTTGATCTGGTCGCTGTC-3′; IL-1β were 5′-CTCCACCTCCAGGGACAGGATATG-3′ and 5′-TCATCTTTCAACACGCAGGACAGG-3′; TNF-α were 5′-TGGCGTGGAGCTGAGAGATAACC-3′ and 5′-CGATGCGGCTGATGGTGTGG-3′; IL-4 were 5′-TCTCACCTCCCAACTGCTTCCC-3′ and 5′-TCTGCTCTGTGAGGCTGTTCAAAG-3′; IL-10 were 5′-GCTGAGAACCAAGACCCAGACATC-3′ and 5′-AAAGGCATTCTTCACCTGCTCCAC-3′; IL-6 were 5′-GAGTAGTGAGGAACAAGCCAGAGC-3′ and 5′-GTTGGGTCAGGGGTGGTTATTGC-3′.

### 4.13 Immunofluorescent staining

To examine changes in the expression of key proteins, double immunostaining was performed. In summary, samples are then incubated with primary antibodies to the corresponding proteins before being incubated with secondary antibodies. The secondary antibodies will be labeled with Alexa Fluor® 594 and Alexa Fluor® 488, respectively. Subsequently, DAPI staining solution was applied to the slides. The image capture process involved the utilization of fluorescence microscopy.

### 4.14 siRNA transfection

Mt1gene knockdown was performed using siRNA transfection. Briefly, WPMY-1 cells were seeded in 6-well plates and cultured until reaching 60-70% confluence. Cells were then transfected with 100 nM of either Mt1-specific siRNA or negative control siRNA using Lipofectamine 3000 reagent according to the manufacturer's protocol. The culture medium was replaced with fresh complete medium 6 h post-transfection. The efficiency of Mt1knockdown was validated at protein levels by Western blotting, respectively, at 48 h after transfection. For the functional assay, the transfected cells were treated with QM(Zn) NPs for 24 h before subsequent protein extraction and Western blot analysis.

### 4.15 Western blotting analysis

The alterations in protein expression were assessed more visually using Western blotting experiments. First, total protein was extracted from the tissue using RIPA lysis buffer. Then, the protein concentration was quantified using the BCA kit after denaturing the proteins by adding loading buffer. The total protein was subjected to electrophoresis, followed by transfer to a polyvinylidene difluoride (PVDF) membrane. The PVDF membrane is then subjected to a gradual incubation process with primary and secondary antibodies. The visualization of protein bands is achieved through the use of electrochemiluminescence (ECL) light-emitting solutions.

### 4.16 Multi-omics analysis

Samples were subjected to a process of grinding in liquid nitrogen, followed by lysis with SDT and DTT. Subsequently, the samples were sonicated on ice for a duration of 5 min. Following this, the samples undergo a cooling process to 4 °C, during which an ice bath is utilized for a duration of 2 min. Subsequently, the samples are subjected to a centrifugation step at 12,000g for a duration of 15 min at 4 °C. This is followed by the alkylation of the samples with IAM in a dark environment. Subsequently, the samples undergo thorough mixing with pre-cooled acetone, followed by storage at −20 °C for a duration of 2 h. Subsequent to the use of cold acetone for the purpose of washing, the precipitate is dissolved in DB buffer. Subsequently, trypsin and TEAB buffer are added, and the sample is digested at 37 °C for 4 h. In order to execute this procedure, it is first necessary to mix formic acid with the sample. Then, the pH must be adjusted to below 3. Next, the sample must be subjected to centrifugation at 12,000 g for 5 min at room temperature. The subsequent step involves the application of a C18 desalting column, followed by a series of washes with Wash Buffer and an elution process with Elution Buffer. The eluate from each sample was collected and lyophilized. The analysis was conducted using a mass spectrometer.

The mRNA was purified from total RNA using poly T oligonucleotide ligated magnetic beads. The fragmentation process was executed within the context of a first strand synthesis reaction buffer (5X), with the utilization of divalent cations at elevated temperatures. First-strand cDNA was synthesized using random hexamer primers and M-MuLV reverse transcriptase. Subsequent to this, second-strand cDNA synthesis was performed using DNA polymerase I and RNase H. Thereafter, remaining overhanging ends were converted to blunt ends by exonuclease/polymerase activity. Subsequent to the adenylation of the 3′ end of the DNA fragment, the adapter with a hairpin loop structure was ligated in preparation for hybridization. The 370-420 bp cDNA fragments were purified and selected using the AMPure XP System. Subsequently, they were subjected to PCR using Phusion High Fidelity DNA Polymerase, universal PCR primers, and Index (X) primers. The final step in the procedure was the purification of the PCR products, followed by an assessment of the library quality using an Agilent Bioanalyzer 2100 System.

### 4.17 Statistical analysis

All data are expressed as mean ± standard deviation (SD). The significance of the results was assessed using GraphPad Prism version 9.5 software with one-way ANOVA, with a significance threshold of p < 0.05. In cases where the data did not follow a normal distribution, Fisher's exact test was used to calculate the results.

## Ethics approval and consent to participate

All animal experiments complied with the ARRIVE guidelines and were carried out in accordance with the Animals (Scientific Procedures) Act 1986.

All animal experiments complied with the Guide for the Care and Use of Laboratory Animals of the Ethical Committee of Xi'an Jiaotong University, Xi'an, China (permit No. XJTU 2019–003).

## CRediT authorship contribution statement

**Kailai Liu:** Conceptualization, Methodology, Project administration, Writing – original draft. **Yuchen Zhang:** Formal analysis, Methodology, Project administration, Writing – original draft. **Yanyao Gao:** Formal analysis, Methodology, Project administration. **Yunhe Zheng:** Data curation, Methodology, Supervision. **Yu Huang:** Formal analysis, Methodology, Validation. **Jiangchuan He:** Investigation, Methodology, Software. **Ting Wang:** Data curation, Investigation, Validation. **Hanchao Zhou:** Data curation, Supervision. **Jinpeng Wen:** Data curation, Validation. **Zhengye Sun:** Methodology, Visualization. **Keke Wang:** Data curation, Investigation, Software. **Qiang Fu:** Formal analysis, Project administration. **Bin Song:** Formal analysis, Visualization. **He Wang:** Formal analysis, Methodology. **Lei Wang:** Funding acquisition, Investigation, Validation, Writing – review & editing. **Geng Zhang:** Conceptualization, Funding acquisition, Writing – review & editing. **Ke Wang:** Funding acquisition, Investigation, Validation, Writing – review & editing.

## Declaration of competing interest

The authors declare that they have no known competing financial interests or personal relationships that could have appeared to influence the work reported in this paper.
