# Supplementary material for: Reversing zinc dyshomeostasis and oxidative stress: A metallothionein 1-activating MXene/Zn2+ triple-action nanotherapeutic strategy for chronic non-bacterial prostatitis
Source: Bioact Mater. 2026 Aug 21;67:656–75. doi: 10.1016/j.bioactmat.2026.07.037 (PMC13524627; doi:10.1016/j.bioactmat.2026.07.037)
Supplement: Supplementary file 1 [file mmc1.docx]

**Reversing Zinc Dyshomeostasis and Oxidative Stress: A Metallothionein 1-Activating MXene/Zn^2+^ Triple-Action Nanotherapeutic Strategy for Chronic Non-Bacterial Prostatitis**

**Figure S1** The the ^1^H NMR spectra of QZ@Chs and Chs.

**Figure S2** The UV-Vis spectra of Ti_3_C_2_ MXene.

**Figure S3** The C, O, S, and N high-resolution spectra of QM (Zn) NPs.

**Figure S4** The TEM-EDS mapping images of QM (Zn) NPs. Scale bar = 500 nm.

**Figure S5** The representative images of stability experiment.

**Figure S6** The ONOO^-^ scavenging ratios of QM (Zn) NPs. n = 3. All values are expressed as mean ± SD.

**Figure S7** The O_2_^-^ scavenging ratios of QM (Zn) NPs. n = 3. All values are expressed as mean ± SD.

**Figure S8** The cell viability of L 929 cells treated with QM (Zn) NP. n = 3. All values are expressed as mean ± SD.

**Figure S9** The representative live/dead staining of RAW264.7 cells after incubation with QM (Zn) NP. Scale bar = 100 μm.

**Figure S10** The representative live/dead staining of L 929 cells after incubation with QM (Zn) NP. Scale bar = 100 μm.

**Figure S11** The representative Masson staining images of major organs of rats treated with QM (Zn) NPs. Scale bar = 400 μm.

**Figure S12** The representative H&E and Masson staining images of major organs of rats treated with QM (Zn) NPs for 90 days. Scale bar = 400 μm.

**Figure S13** The cellular uptake experimental treatment protocols.

**Figure S14** Statistical results of macrophage polarization flow data. n = 3. All values are expressed as mean ± SD. ****P*<0.001, ###*P*<0.001, vs LPS.

**Figure S15** The flow assay for macrophage polarization (treatment with QM (Zn) NPs alone).

**Figure S16** The representative NIR fluorescence pictures of prostatitis rats with Cy7 at 6h after intravenous injection.

**Figure S17** Pharmacokinetic profile of QM(Zn) NPs in rats. Data are presented as mean ± SD (n = 3).

**Figure S18** Statistical Results of ROS Fluorescence Intensity in WPMY-1 cells. n = 3. All values are expressed as mean ± SD. ****P*<0.001.

**Figure S19** Statistical results of JC-1 staining fluorescence intensity in WPMY-1 cells. n = 3. All values are expressed as mean ± SD.

**Figure S20** The expression of IL-4 mRNA in the WPMY-1 cells. n = 3. All values are expressed as mean ± SD. ****P*<0.001, *****P*<0.0001.

**Figure S21** The therapeutic efficacy of QM (Zn) NPs in non-bacterial prostatitis. **A**, The weight index in various groups. n = 3. **B**, The representative thymus and spleen images of rats in various groups. **C**, The prostate index in various groups. n = 3. **D**, The spleen index in various groups. n = 3. **E**, The thymus index in various groups. n = 3. **F**, The representative H&E and Masson staining images of prostates in rats under different treatments. Scale bar = 400 μm. All values are expressed as mean ± SD. Statistical significance was determined using a one-way ANOVA with Tukey’s multiple comparisons. **P* < 0.05, ***P* < 0.01.

**Figure S22** The representative images of immunofluorescence staining in non-bacterial prostatitis rats. Scale bar = 400 μm.

**Figure S23** Measurement of pH in the prostate tissue. Data are presented as mean ± SD (n = 3). ***P < 0.001 versus the Control group, determined by an unpaired t-test.

**Figure S24** The representative prostate images of rats in various groups.

**Figure S25** The EDS elemental analysis results of SEM of prostate tissue.

**Figure S26** The expression of IL-17A, IL-4 and SP in prostates of rats under different treatments. All values are expressed as mean ± SD. Statistical significance was determined using a one-way ANOVA with Tukey’s multiple comparisons. n = 3. **P*<0.05, ****P* < 0.001.

**Figure S27** The comparison of therapeutic efficacy of QM (Zn) NPs and celecoxib in CNP. **A**, The weight index in various groups. n = 3. **B**, The representative prostate, thymus and spleen images of rats in various groups. **C**, The prostate index in various groups. n = 3. **D**, The thymus index in various groups. n = 3. **E**, The spleen index in various groups. n = 3. **F,** The Zn content in various groups. n=3. **G**, The representative H&E and Masson staining images of prostates in rats under different treatments. Scale bar = 400 μm. All values are expressed as mean ± SD. Statistical significance was determined using a one-way ANOVA with Tukey’s multiple comparisons. **P* < 0.05, ****P* < 0.001.

**Figure S28** The KEGG pathway enrichment analysis in Model and Control groups. n = 3.

**Figure S29** The proteomics PCA plot of Control, Model, and QM (Zn) NPs groups. n = 3.

**Figure S30** The Venn diagram of all differential proteins’ expression for comparisons between groups. n = 3.

**Figure S31** The proteomics volcano plot.

**Figure S32** The heatmap of representative differential expressed proteins in three groups.

**Figure S33** The expression of IκB mRNA in the prostate tissue. n = 3. All values are expressed as mean ± SD. Statistical significance was determined using a one-way ANOVA with Tukey’s multiple comparisons.

**Figure S34** The representative fluorescent images of expression of Timp1 and Mmp9 in prostates under different treatments. Scale bar = 400 μm.

**Figure S35** The fluorescence intensity statistics of Timp1 and Mmp9. n = 3. All values are expressed as mean ± SD. Statistical significance was determined using a one-way ANOVA with Tukey’s multiple comparisons. ****P*<0.001, ###*P* < 0.001, vs Model.

**Figure S36** The gray scale analysis results of WB images. n = 3.

**Figure S37** The expression of Mmp9 mRNA in the prostate tissue. n = 3. All values are expressed as mean ± SD. Statistical significance was determined using a one-way ANOVA with Tukey’s multiple comparisons. **P*<0.05, ***P*<0.01.

**Figure S38** The expression of Timp1 mRNA in the prostate tissue. n = 3. All values are expressed as mean ± SD. Statistical significance was determined using a one-way ANOVA with Tukey’s multiple comparisons. *****P*<0.0001.

**Figure S39** The expression of TNF-α, IL-10 and IL-4 mRNA in the prostate tissue. n = 3. All values are expressed as mean ± SD. Statistical significance was determined using a one-way ANOVA with Tukey’s multiple comparisons. ****P*<0.001, *****P*<0.0001

**Figure S40** The representative WB images of expression of Mmp9, Timp1, Mgst1, Ptx3, and Muc1 protein in the WPMY-1 cells. n = 3.

**Figure S41** The expression of Mmp9 mRNA in the WPMY-1 cells. n = 3. All values are expressed as mean ± SD. Statistical significance was determined using a one-way ANOVA with Tukey’s multiple comparisons. ***P*<0.01.

**Figure S42** The expression of Timp1 mRNA in the WPMY-1 cells. n = 3. All values are expressed as mean ± SD. Statistical significance was determined using a one-way ANOVA with Tukey’s multiple comparisons. ***P*<0.01, ****P*<0.001.

**Figure S43** The expression of Mgst1 mRNA in the WPMY-1 cells. n = 3. All values are expressed as mean ± SD. Statistical significance was determined using a one-way ANOVA with Tukey’s multiple comparisons. **P*<0.05, ***P*<0.01.

**Figure S44** The expression of Ptx3 mRNA in the WPMY-1 cells. n = 3. All values are expressed as mean ± SD. Statistical significance was determined using a one-way ANOVA with Tukey’s multiple comparisons. ***P*<0.01.

**Figure S45** The expression of MUC1 mRNA in the WPMY-1 cells. n = 3. All values are expressed as mean ± SD. Statistical significance was determined using a one-way ANOVA with Tukey’s multiple comparisons. ****P*<0.001, *****P*<0.0001.

**Figure S46** The representative WB images of expression of Mt1, IKK, IκB, p-IκB, p65 and p-p65 protein in the WPMY-1 cells with treated by siRNA and QM(Zn). n = 3.

**Figure S47** The detection of intracellular zinc ion content. n = 3, All values are expressed as mean ± SD. Statistical significance was determined using a one-way ANOVA with Tukey’s multiple comparisons. ***P < 0.01, ***P < 0.001*.

**Figure S48** The ability to protect neuronal cells. **A**, The representative live/dead staining of HMC3 cells after incubation with QM (Zn) NPs. Scale bar = 200 μm. **B**, The cell viability of SH-SY5Y cells treated with QM (Zn) NPs. n = 3. **C**, The representative ROS staining images of SH-SY5Y under t-BOOH and QM (Zn) NPs treatment. Scale bar = 200 μm. **D**, The expression of TAC1 mRNA in the prostate tissue. n = 3. **E**, The expression of IL-1β mRNA in the prostate tissue. n = 3. **F**, The expression of TNF-α in the prostate tissue. n = 3. All values are expressed as mean ± SD. Statistical significance was determined using a one-way ANOVA with Tukey’s multiple comparisons. **P* < 0.05, ** *P* < 0.01.
